# The Efficacy of Probiotics, Prebiotics, Synbiotics, and Fecal Microbiota Transplantation in Irritable Bowel Syndrome: A Systematic Review and Network Meta-Analysis

**DOI:** 10.3390/nu16132114

**Published:** 2024-07-02

**Authors:** Youhe Wu, Yuetong Li, Qi Zheng, Lanjuan Li

**Affiliations:** State Key Laboratory for Diagnosis and Treatment of Infectious Diseases, National Clinical Research Center for Infectious Diseases, National Medical Center for Infectious Diseases, Collaborative Innovation Center for Diagnosis and Treatment of Infectious Diseases, The First Affiliated Hospital, Zhejiang University School of Medicine, 79 Qingchun Rd., Hangzhou 310003, China

**Keywords:** irritable bowel syndrome, probiotic, fecal microbiota transplantation, network meta-analysis, prebiotic, synbiotic

## Abstract

Irritable bowel syndrome (IBS) is a common gastrointestinal disorder with gut microbiota imbalance playing a significant role. There are increasing numbers of research studies exploring treatment options involving probiotics, prebiotics, synbiotics, and fecal microbiota transplantation (FMT), but it is still uncertain which treatment option is superior. The research was conducted on various databases and unpublished trial data (up to February 2023). Randomized controlled trials (RCTs) were screened for adult patients with IBS comparing interventions with placebo. Probiotics, prebiotics, synbiotics, and FMT were assessed for their impact using mean difference and Bayesian network meta-analysis. Out of 6528 articles, 54 were included for probiotics, 7 for prebiotics/synbiotics, and 6 for FMT. Probiotics showed improvement in IBS symptoms, particularly with *Bifidobacterium* and *Lactobacillus* strains. Prebiotics and synbiotics did not show significant improvement. Network meta-analysis indicated the favorable effects of probiotics (OR = 0.53, 95% CI, 0.48 to 0.59) and FMT (OR = 0.46, 95% CI, 0.33 to 0.64) on IBS, with no serious adverse events reported. In short, probiotics and FMT are effective for managing IBS, with *Bifidobacterium* and *Lactobacillus* being dominant strains. However, the most effective probiotic combination or strain remains unclear, while prebiotics and synbiotics did not show significant improvement.

## 1. Introduction

Irritable bowel syndrome (IBS) is a common functional gastrointestinal disorder, with a global prevalence ranging from 1.1% to 45%. The prevalence in most Western countries and China is between 5% and 10%. Although the prevalence fluctuates, it is not steadily increasing [1]. Notably, within the affected demographic, the likelihood of women having IBS is 1.5 to 3.0 times higher than that of men, with prevalence rates of 8.9% in men and 14% in women [2]. IBS is characterized by recurrent abdominal pain, bloating, and abnormal bowel habits. Currently, it is defined based on symptom criteria, without detectable anatomical, inflammatory, or biochemical pathological changes [3]. Due to its recurrent and chronic nature, IBS significantly affects patients’ quality of life and imposes a substantial socio-economic burden [4].

The pathophysiological mechanisms of IBS are not yet fully understood, with various factors contributing to the occurrence and progression of the disease, including the superficial mucosal inflammation of the colon, impaired epithelial barrier function, mucosal immune activation, the dysbiosis of the gut microbiota, psychological stress, and the dysregulation of the brain–gut–microbiota axis [5,6]. The levels of cytokines (such as TNF-α and IL-6) and chemokines (such as CXCL9 and CXCL10) differ between patients with IBS and healthy individuals, and the severity of inflammation correlates with the severity of visceral hypersensitivity. High concentrations of cytokines are also associated with anxiety and depression in patients with diarrhea-predominant IBS (IBS-D) [7,8]. In addition, increased intestinal permeability can lead to mild immune cell infiltration of the intestinal mucosa, which is considered an early event in IBS [9]. Studies have also shown that increased intestinal permeability is associated with the severity of diarrhea and pain, which may play a role in the development of IBS symptoms [10,11]. Furthermore, stress-induced changes in the neuroendocrine–immune pathways affect the gut–brain axis and the microbiota–gut–brain axis, leading to the onset or exacerbation of IBS symptoms [12]. After stress induction, mast cells, intestinal chromaffin cells, and lymphocytes are activated, which are the main immune cells in the intestine. They secrete proteases, 5-hydroxy tryptamine (5-HT), and proinflammatory cytokines, leading to mucosal immune dysregulation [13,14]. Histamine, 5-HT, and proteases secreted in the intestine can activate enteric nervous system (ENS) neurons, which may be a potential mechanism for visceral hypersensitivity in IBS [15,16]. Furthermore, more and more studies suggest that in IBS, abnormalities in the hypothalamic–pituitary–adrenal (HPA) axis, immune system, gut–brain axis, and ENS are caused by abnormalities in the epithelial barrier, gut microbiota, and bile acids [11].

The gut microbiota is diverse and complex, playing a complex and important role in maintaining host ecosystem balance and normal physiological function [17]. Multiple studies have shown significant differences in gut microbiota between patients with IBS and healthy control groups, and the dysbiosis and reduced diversity of gut microbiota are associated with the development of IBS [18,19,20]. Patients with IBS have an increased abundance of *Enterobacteriaceae*, *Streptococcus*, *Clostridium*, *Gemella*, and *Ruminococcus*, while beneficial bacteria such as *Roseburia* and *Faecalibacterium* are decreased [21]. The composition and activity of *Bifidobacterium* are low in both the fecal and mucosal samples of patients with IBS [22]. Patients with IBS-D have a decreased abundance of fecal microbiota, with an increase in *Proteobacteria* and a decrease in *Firmicutes* [23]. However, other studies have shown a decrease in *Firmicutes* and an increase in *Proteobacteria* in IBS [24]. Currently, there is no consensus on the gut microbiota in IBS, possibly due to the lack of control for factors such as age, gender, race, diet, and antibiotic intake in the included studies, and the subtyping of IBS based on microbial characteristics has not been established [25]. However, studies showed that some probiotics appear to have anti-inflammatory effects and can alleviate visceral hypersensitivity in IBS [26,27,28]. The Rome team has indicated that there is sufficient evidence to show that the gut microbiota of patients with IBS is disturbed, and dietary management, probiotics, prebiotics, and synbiotics are promising approaches for managing IBS [29].

Conventional treatments for IBS primarily target symptom management, with interventions such as loperamide for diarrhea or laxatives for constipation offering limited efficacy in addressing symptoms like bloating and abdominal pain [30]. In recent years, various treatment methods, such as a low FODMAP diet, antihistamines (e.g., ebastine), serotonin receptor agents (e.g., ondansetron and prucalopride), ileal bile acid transporter agents (e.g., elobixibat), and gut–brain neuromodulators (e.g., pregabalin), have been proven to be effective. Additionally, more and more research and clinical evidence emphasize the importance of the gut microbiota in the pathophysiology of IBS, making probiotics an increasingly important treatment option [31]. Probiotics, prebiotics, non-absorbable antibiotics, and fecal microbiota transplantation (FMT) have shown positive effects in relieving IBS symptoms by addressing gut dysbiosis. However, the long-term use of non-absorbable antibiotics may lead to antimicrobial resistance, while probiotics, prebiotics, and FMT do not pose resistance issues [32].

Although numerous studies have shown the efficacy of probiotics in treating IBS, it is still unknown which specific probiotic species, strains, or combinations of probiotics are more optimal [33]. Additionally, an increasing body of research suggests that FMT can effectively alleviate IBS symptoms, but whether it is superior to probiotics, prebiotics, and synbiotics requires further investigation [34]. Therefore, in this study, we conducted a network meta-analysis to compare the therapeutic effects of probiotics, prebiotics, synbiotics, and FMT on IBS. We aimed to explore the optimal intervention for gut microbiota and provide better treatment options for clinical practice, as well as directions for further research in IBS treatment.

## 2. Materials and Methods

### 2.1. Search Strategy and Selection Criteria

We searched the MEDLINE (from 1946 to February 2023), EMBASE and EMBASE Classic (from 1947 to February 2023), Cochrane Central Register of Controlled Trials, and Web of Science databases. In addition, we searched for unpublished trial data in clinicaltrials.gov (www.ClinicalTrials.gov/, accessed on 23 February 2023). The search strategy is detailed in the Supplementary Materials (Search Strategy). We also manually searched conference proceedings (Digestive Disease Week, American College of Gastroenterology, United European Gastroenterology Week, and Asia Pacific Digestive Week) from 2001 to 2023 to identify trial results that were available only in abstract format. Finally, we tracked the reference lists of relevant published clinical trials and reviews.

Randomized controlled trials (RCTs) that include patients with IBS aged ≥18 years and meeting the diagnostic criteria for IBS (including Rome criteria I, II, III, IV, and Manning criteria) are eligible for inclusion in our study. The eligible interventions include the use of single or combination probiotics, prebiotics, synbiotics, or FMT, with a placebo as the control intervention. The minimum duration of treatment is seven days (with one or more treatments for FMT), with a follow-up period of at least seven days. The outcome measures of interest include whether overall IBS symptoms improved or not (dichotomous outcome), overall or individual IBS symptom scores (including abdominal pain, bloating, etc.), and IBS quality of life (IBS-QoL) score, as well as trial-related adverse events. Trials reporting these data are eligible for inclusion. There is no language restriction for the language of publication.

Two authors (Youhe Wu and Yuetong Li) independently screened the titles and abstracts of studies, excluding duplicates and studies that were not related to the topic. After that, full-text articles were read for a second screening, and the studies that did not meet the inclusion criteria or had incomplete data were excluded. Disagreements were resolved through consensus. Prior to the implementation of the study, we registered the study in the PROSPERO international prospective register of systematic reviews (registration number: CRD42023408698).

### 2.2. Outcome Assessment

The primary outcome is the evaluation of overall IBS symptoms. Data types include dichotomous variable and continuous variable, where the dichotomous variable is whether overall symptoms improve or not after intervention, and the continuous variable is the difference in overall symptom scores before and after intervention (such as IBS symptom severity scale (IBS-SSS) and the Francis severity score questionnaire). Secondary outcomes include scores (such as the Likert scale) for individual IBS symptoms (such as abdominal pain and bloating) after intervention and quality of life scores (such as IBS-QoL score). Adverse events are also included in the analysis.

### 2.3. Data Extraction

Two authors (Youhe Wu and Yuetong Li) independently extracted the data to a Microsoft Excel spreadsheet (version 2019, Microsoft, Redmond, WA, USA), which was then checked and reviewed by a third author (Qi Zheng). We extracted the dichotomous outcome of whether the overall IBS symptoms improved or not. For continuous outcomes, we extracted the mean and standard deviation (SD) of the changes from baseline in the overall or individual symptom scores within each group. For the studies where the mean changes from baseline were not reported, we extracted the post-intervention mean and SD of the overall or individual symptom scores. For the studies that did not report SDs, we used the Cochrane RevMan calculator (https://training.cochrane.org/resource/revman-calculator, accessed on 23 February 2023) to estimate. If a study involved multiple follow-ups, we chose the data recorded at the last follow-up as the outcome data. For clinical characteristics, we extracted the author, publication year, study duration, country, diagnostic criteria, subject characteristics (including the number of cases, male–female ratio, and number of IBS subtypes), intervention types (probiotics, prebiotics, synbiotics, FMT), and specific strains, as well as the treatment dosage and frequency, treatment duration, and follow-up time.

### 2.4. Quality Assessment and Risk of Bias

Two authors (Youhe Wu and Yuetong Li) independently assessed the risk of bias (selection, performance, detection, follow-up survey, and reporting bias) of the included trials using the Cochrane risk of bias tool. Disagreements were resolved through discussion and consensus. We meticulously documented several key domains to assess the risk of bias within the included studies. These domains comprised the random sequence generation and allocation concealment measures, blinding of implementation and outcome assessment, completeness of outcome data, and consistency between reported and expected results. Each domain was rated as either “low risk”, “high risk”, or “unclear” based on predefined criteria. The overall risk of bias for each study was determined by the most serious risk observed across all the domains. When three or more domains had “unclear” risk, the study was considered to have a high risk of bias [35].

### 2.5. Data Synthesis and Statistical Analysis

We used standardized mean differences (SMDs) to evaluate the comparative effect sizes between the experimental groups (probiotics, prebiotics, synbiotics, and FMT) and the placebo group. We assumed heterogeneity existed among the studies and used a random effects model to analyze the data. Specifically, in the meta-analysis of the overall assessment of IBS symptoms, we converted odds ratios (ORs) to SMD using the formula $SMD=\frac{\sqrt{3}}{\pi }\ln OR$, based on the assumption of a logistic distribution for continuous measurements within each intervention group and the same variability between the intervention and control groups [36,37]. We converted the dichotomous outcome of overall symptom maintenance or no improvement into SMD to reduce the number of studies excluded from the analysis. Furthermore, we separately collected the mean and SD of the changes from baseline in the overall symptoms for probiotics, prebiotics, synbiotics, and FMT to explore their effects on improving IBS symptoms. Individual symptom effects were also analyzed for each treatment approach. Additionally, we further investigated the effects of different probiotic strains and mixed strains on improving IBS symptoms by dividing probiotic interventions into multiple subgroups based on different strains.

Thereafter, we conducted a Bayesian network meta-analysis to explore the indirect comparison of the effects of probiotics, prebiotics, synbiotics, and FMT on IBS. We used pooled relative risks (RRs) (with 95% confidence intervals (95%CI)) as the effect size for comparing the different treatments, with a random effects model for conservative estimation. The primary outcome of interest in our analysis was the absence of improvement in clinical symptoms. If the 95%CI did not cross the value of 1, the statistical significance between the two groups was considered to be significant.

Since the standard deviation of pre–post differences and the standard deviation of post-intervention have different meanings, it is not appropriate to directly combine the pre–post-intervention changes and post-intervention data. For the studies that simultaneously included baseline and post-intervention measurement values, we estimated the standard deviation of the pre–post-intervention differences. Additionally, since pooling the SMD and SD of the pre–post-intervention changes is more likely to yield positive results, we also performed a sensitivity analysis by pooling the SMD and SD of the post-intervention measurements for both overall and individual symptoms.

Statistical analyses were conducted using R (v4.3.1). We used the combination of *I*^2^, Cochran’s Q test, and τ^2^ values to assess heterogeneity. Heterogeneity was considered low when *I*^2^ was not higher than 50% and *p* > 0.1 [38]. We performed Egger’s test to examine possible publication bias. Both *meta* and *metafor* packages were used to conduct the meta-analysis of the primary and secondary outcomes for probiotics, prebiotics, synbiotics, and FMT. The *gemtc* package was used to perform the Bayesian network meta-analysis for the indirect comparison of the four treatment approaches. Finally, *forestplot* package was used to create the forest plots for RRs and SMDs.

## 3. Results

**Figure 1:**
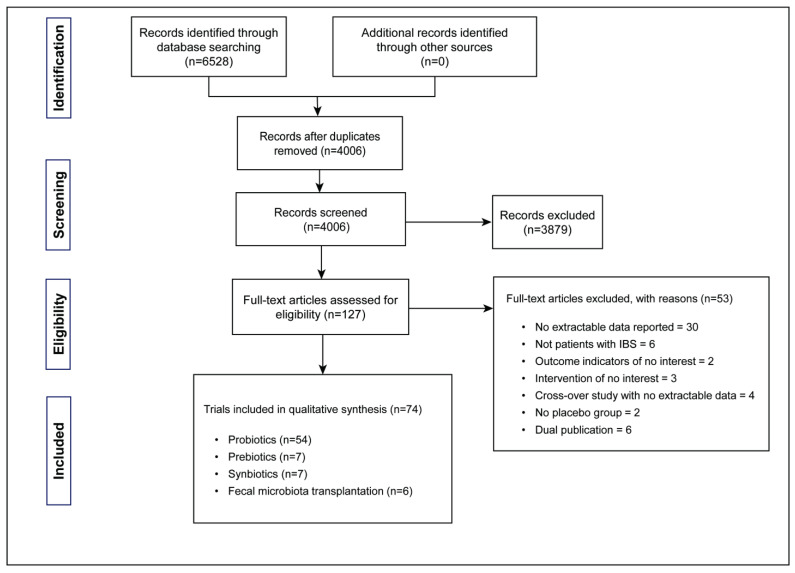
Flow diagram of study selection in the systematic review and meta-analysis.

We identified a total of 6528 articles through our search, and 127 articles were found to be relevant to our study after screening. Among them, 53 articles were excluded for various reasons, and the remaining articles included 54 on probiotics, 7 on prebiotics and synbiotics, and 6 on FMT (Figure 1).

### 3.1. Efficacy and Safety of Probiotics

A total of 54 double-blind RCTs on probiotic treatment were included, involving a total of 6732 participants (3707 in the intervention group) (Supplementary Table S1). Of these, 36 trials were assessed as having a low risk of bias. Among the included trials, 22 trials involved a mixture of probiotics, 11 trials involved *Lactobacillus* or *Lactobacillus* combinations, 9 trials involved *Bifidobacterium* or *Bifidobacterium* combinations, 5 trials involved *Bacillus*, and 6 trials involved *Saccharomyces*. Additionally, there was one trial each involving *Clostridium* and *Escherichia. coli*.

#### 3.1.1. Probiotics Can Effectively Improve Overall Symptoms of IBS

**Figure 2:**
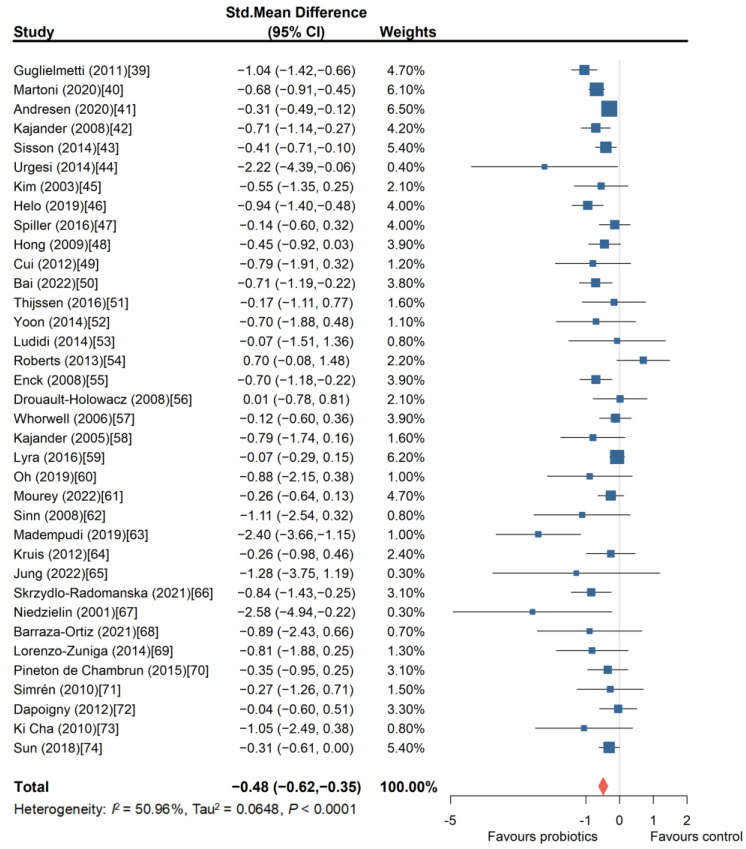
Forest plot of randomized controlled trials of probiotics versus placebo in irritable bowel syndrome: effect on global symptom change from baseline [39,40,41,42,43,44,45,46,47,48,49,50,51,52,53,54,55,56,57,58,59,60,61,62,63,64,65,66,67,68,69,70,71,72,73,74].

A total of 36 RCTs [39,40,41,42,43,44,45,46,47,48,49,50,51,52,53,54,55,56,57,58,59,60,61,62,63,64,65,66,67,68,69,70,71,72,73,74] compared probiotics with placebo, involving 5445 patients, with 3038 in the intervention group. We conducted a meta-analysis of the overall symptom improvement effect sizes of these studies. The results indicated that probiotics significantly improved the overall symptoms of patients with IBS (SMD = −0.48, 95% CI, −0.62 to −0.35) (Figure 2). However, the funnel plot displayed an asymmetrical distribution of the included data (Supplementary Figure S1), suggesting potential publication bias or effects from small sample studies (Egger’s test, *p* = 0.0229). Therefore, as a sensitivity analysis, we also combined the effect sizes of overall symptom scores post-intervention, and the results remained consistent. Probiotics showed a statistically significant improvement in the overall symptoms of IBS compared to placebo (SMD = −0.41, 95% CI, −0.59 to −0.23).

**Figure 3:**
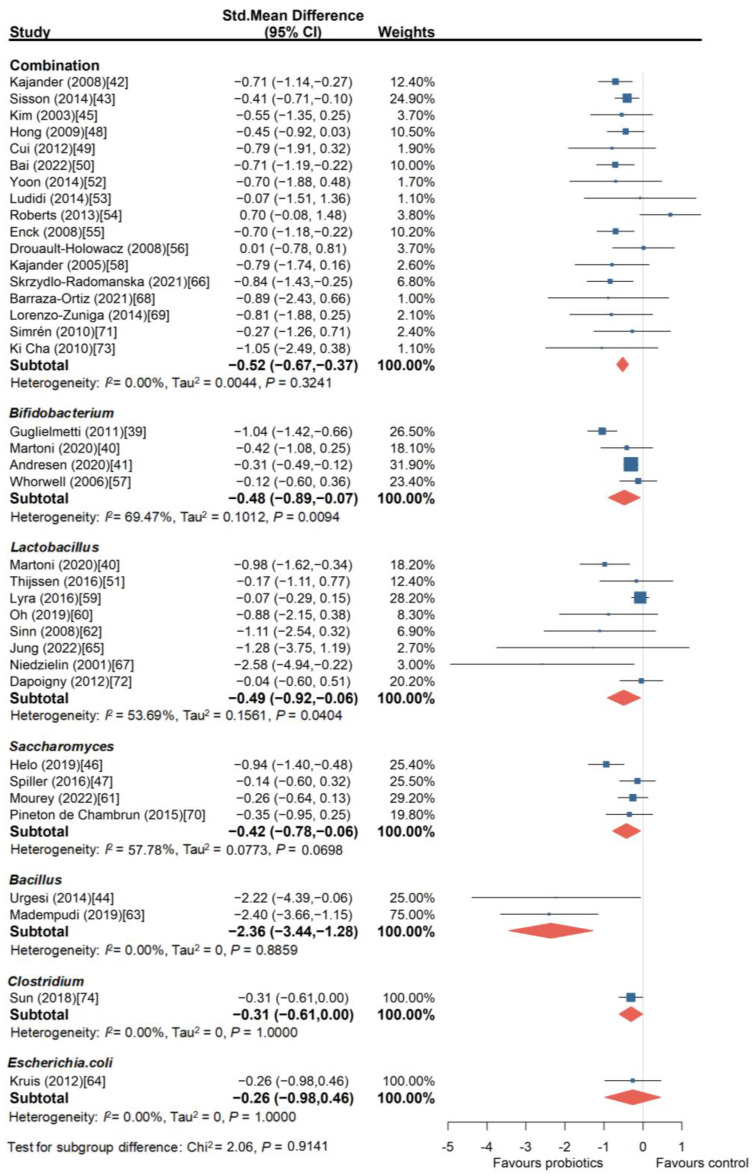
Forest plot of randomized controlled trials of probiotics versus placebo in irritable bowel syndrome: effect on global symptom change from baseline [39,40,41,42,43,44,45,46,47,48,49,50,51,52,53,54,55,56,57,58,59,60,61,62,63,64,65,66,67,68,69,70,71,72,73,74].

To further explore which probiotic species, strains, and combination approaches are more beneficial for improving IBS symptoms, we conducted subgroup analyses of different probiotic combinations and species. As shown in Figure 3, the meta-analysis of the trials involving probiotic combinations showed a beneficial effect on overall symptom improvement in IBS (SMD = −0.52, 95% CI, −0.67 to −0.37). However, two trials involving the combination of *Bifidobacterium lactis* CNCM I-2494, *Streptococcus thermophilus*, and *Lactobacillus bulgaricus* [54] (SMD = 0.70, 95% CI, −0.08 to 1.48) and the combination of *Bifidobacterium longum* LA 101, *Lactobacillus acidophilus* LA 102, *Lactococcus lactis* LA 103, and *Streptococcus thermophilus* LA 104 [56] (SMD = 0.01, 95% CI, −0.78 to 0.81) did not show a significant benefit in improving overall IBS symptoms. There was no publication bias among these studies (Egger’s test, *p* = 0.9806). In addition, the sensitivity analysis reaffirmed the significant beneficial effect of probiotic combinations on overall IBS symptoms (SMD = −0.41, 95% CI, −0.62 to −0.19).

There were four *Bifidobacterium* intervention trials [39,40,41,57] involving a total of 1094 patients, and they also showed improvement in the overall symptoms of IBS (SMD = −0.48, 95% CI, −0.89 to −0.07). *Lactobacillus* was used in eight RCTs [40,51,59,60,62,65,67,72] involving 846 patients, and all the trial results suggested improvement in the overall symptoms of IBS (SMD = −0.49, 95% CI, −0.92 to −0.06). There were four RCT [46,47,61,70] trials using *Saccharomyces* (involving 1320 patients), and all of them used the *Saccharomyces cerevisiae* I-3856 strain, which showed improvement compared to placebo (SMD = −0.42, 95% CI, −0.78 to −0.06). Additionally, in two trials comparing *Bacillus* with placebo [44,63] *Bacillus coagulans* (Colinox^®^, Zambon, Milan, Italy.) and *Bacillus coagulans* Unique IS2 strains were used, and both showed beneficial effects on IBS symptoms. Finally, *Clostridium* [74] and *Escherichia. Coli* [64] were each evaluated in one trial, involving 166 and 120 patients, respectively. *Clostridium* seemed to have some improvement effect (SMD = −0.31, 95% CI, −0.61 to 0.00), while *Escherichia. coli* did not show a significant improvement effect (SMD = −0.26, 95% CI, −0.98 to 0.46).

#### 3.1.2. Probiotics Can Effectively Improve Abdominal Pain in Patients with IBS

**Figure 4:**
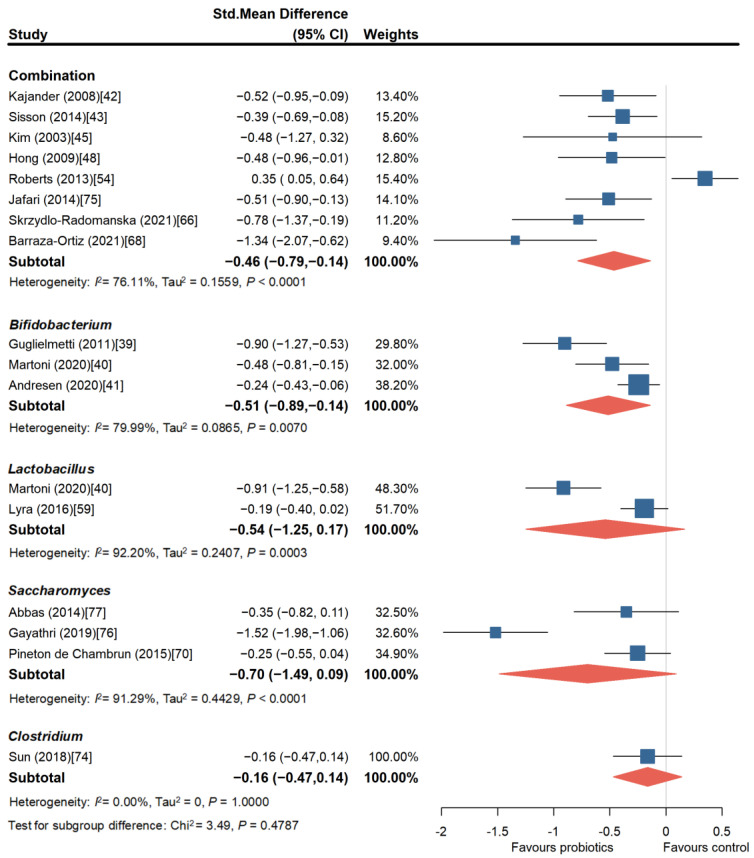
Forest plot of randomized controlled trials of probiotics of different strains versus placebo in irritable bowel syndrome: effect on abdominal pain score change from baseline [39,40,41,42,43,45,48,54,59,66,68,70,74,75,76,77].

To further explore whether probiotics can improve abdominal pain in patients and whether there are more effective probiotic treatment strategies for relieving abdominal pain, we conducted subgroup analyses of abdominal pain symptoms. A total of 16 independent RCT studies involving 2539 patients were analyzed. Among them, eight studies [42,43,45,48,54,66,68,75] evaluated probiotic combinations, and except for two studies [45,54], the rest of the studies all suggested that probiotic combinations significantly alleviated abdominal pain in patients with IBS (SMD = −0.46, 95% CI, −0.79 to −0.14). However, there was publication bias among these studies (Egger’s test, *p* = 0.0205). Three studies [39,40,41] involving *Bifidobacterium* showed that it could alleviate abdominal pain symptoms to a greater extent (SMD = −0.51, 95% CI, −0.89 to −0.14). Only two studies [40,59] evaluating the effect of *Lactobacillus* on abdominal pain were included, and there was significant heterogeneity among the studies, with no significant improvement effect (SMD = −0.54, 95% CI, −1.25 to −0.17). Three studies [70,76,77] evaluated *Saccharomyces*, and the pooled analysis did not find a significant beneficial effect of *Saccharomyces* in relieving abdominal pain, although one study [76] reported a significant alleviation of abdominal pain (SMD = −1.52, 95% CI, −1.98 to −1.06). Lastly, only one study [74] evaluated *Clostridium*, and it was considered to have no effect in relieving abdominal pain (Figure 4).

#### 3.1.3. Probiotics Can Partially Improve Bloating in Patients with IBS

**Figure 5:**
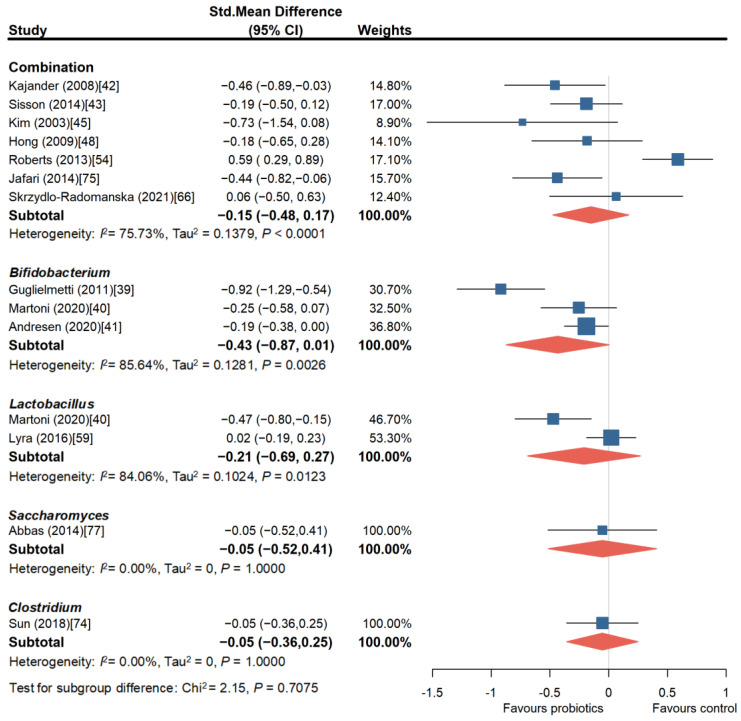
Forest plot of randomized controlled trials of probiotics of different strains versus placebo in irritable bowel syndrome: effect on abdominal bloating score change from baseline [39,40,41,42,43,45,48,54,59,66,74,75,77].

Bloating is also one of the main symptoms in patients with IBS, and we also analyzed the effects of different probiotic interventions on bloating. As shown in Figure 5, seven RCT studies [42,43,45,48,54,66,75] involving 702 patients compared different probiotic strains or combinations with a placebo, and the results showed no statistically significant improvement in bloating (SMD = −0.15, 95% CI, −0.48 to 0.17). However, one study that included a combination of *Lactobacillus rhamnosus*, *Propionibacterium freudenreichii*, *Propionibacterium shermanii*, and *Bifidobacterium* [42], and another study that included a combination of *Bifidobacterium* and *Lactobacillus* [75] suggested a certain improvement in bloating (SMD = −0.46, 95% CI, −0.89 to −0.03 and SMD = −0.44, 95% CI, −0.82 to −0.06). *Bifidobacterium* was studied in three RCTs involving 732 patients, and there was no significant difference in the relief of bloating symptoms between *Bifidobacterium* and placebo (SMD = −0.43, 95% CI, −0.87 to 0.01) [39,40,41]. Two RCTs (involving 560 patients) evaluated *Lactobacillus*, and *Lactobacillus acidophilus* DDS-1 had a better relief effect on bloating compared to placebo (SMD = −0.47, 95% CI, −0.80 to −0.15) [40], while another study comparing *Lactobacillus acidophilus* NCFM (ATCC 700396) with placebo showed no significant relief effect [59]. Finally, in the RCT studies involving *Saccharomyces* and *Clostridium*, there was no significant relief effect on bloating compared to placebo.

#### 3.1.4. Probiotics Can Effectively Improve the Overall Quality of Life in Patients with IBS

**Figure 6:**
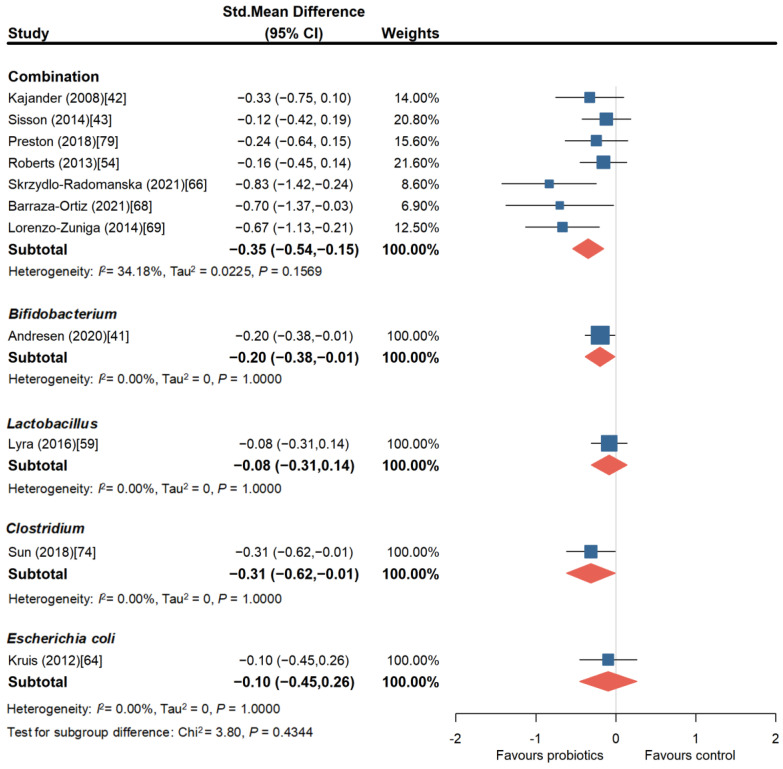
Forest plot of randomized controlled trials of probiotics of different strains versus placebo in irritable bowel syndrome: effect on IBS-QOL score change from baseline [41,42,43,54,59,64,66,68,69,74,79].

The IBS-QoL questionnaire is an important scale for evaluating the quality of life in patients with IBS, with higher scores indicating better IBS-specific quality of life. The IBS-QoL score is related to the patient’s abdominal pain, extraintestinal symptoms, symptom flare-ups, and disease-related issues, and is correlated with disease severity [78]. As shown in Figure 6, probiotic combinations, utilized in seven trials (732 patients) [42,43,54,66,68,69,79], effectively improved the quality of life in patients with IBS (SMD = −0.35, 95% CI, −0.54 to −0.15), with low heterogeneity among the studies (*I*^2^ = 34.18%, *p* = 0.1569). Among them, two RCT studies showed significant improvement in the quality of life in patients with IBS with the probiotic combinations of *Lactobacillus plantarum* (CECT7484 and CECT7485) and *Pediococcus acidilactici* (CECT7483) (SMD = −0.70, 95% CI, −1.37 to −0.03 and SMD = −0.67, 95% CI, −1.13 to −0.21) [68,69]. Only one RCT study (443 patients) involved Bifidobacterium, and it showed a certain improvement in the quality of life compared to the placebo group (SMD = −0.20, 95% CI, −0.38 to −0.01) [41], while in the RCT studies involving Lactobacillus, Clostridium, and Escherichia. coli, there was no significant improvement in the patients’ quality of life scores compared to placebo [59,64,74].

#### 3.1.5. Adverse Events with Probiotics

A total of 433 adverse events involving 5031 study subjects were reported in 54 RCTs. Among them, 244 cases (9.66%) of adverse events were reported in 2527 patients who took probiotics, and 189 cases (8.31%) of adverse events were reported in 2274 subjects who took a placebo. No serious adverse events were reported.

### 3.2. Efficacy and Safety of Prebiotics

A total of seven double-blind RCTs related to prebiotic treatment were included, involving 600 patients (311 in the intervention group) [80,81,82,83,84,85,86]. Five trials were assessed as having a low risk of bias [80,81,83,84,85], while two trials were assessed as having a high risk of bias [82,86].

The results of the seven RCT studies (involving a total of 600 patients) demonstrated no significant difference between the prebiotic and placebo groups in overall symptom improvement (SMD = −0.18, 95% CI, −0.39 to 0.04) [80,81,82,83,84,85,86]. In the assessment of individual symptoms in patients with IBS, three studies reported abdominal pain scores for the prebiotic and placebo groups as continuous variables, and the meta-analysis results indicated no significant difference between the two groups (SMD = 0.42, 95% CI, −2.07 to 2.91) [80,83,85]. In addition, only two studies reported bloating scores for the prebiotic and placebo groups, and the meta-analysis results showed that prebiotics had no significant effect on bloating symptom improvement in patients with IBS (SMD = −0.34, 95% CI, −2.70 to 2.02) [80,85]. Lastly, three studies reported an improvement in the quality of life in patients with IBS, and the results also indicated no significant difference between the prebiotic and placebo groups (SMD = 0.15, 95% CI, −0.10 to 0.39) [82,83,85].

Among the seven RCTs, four studies reported adverse events. Of the 180 patients in the treatment group, 31 (17.22%) experienced adverse events, while in the 171 patients in the placebo group, 28 (16.37%) experienced adverse events. No severe adverse events were reported in any of the trials.

### 3.3. Efficacy and Safety of Synbiotics

A total of seven double-blind RCTs related to synbiotic treatment were included, involving 595 individuals (296 in the intervention group) [87,88,89,90,91,92,93]. One trial was assessed as having a high risk of bias [87], while the remaining six trials were assessed as having a low risk of bias.

The results of five RCTs (involving a total of 511 patients) showed no significant difference between the synbiotic and placebo groups in overall symptom improvement (SMD = −0.01, 95%CI, −0.36 to 0.34) [89,90,91,92,93]. In terms of individual symptoms in patients with IBS, three studies reported abdominal pain scores for the synbiotic and placebo groups as continuous variables, and the meta-analysis results showed that there was no significant difference between the two groups (SMD = −0.93, 95%CI, −3.53 to 1.68) [87,90,92]. Two studies reported an improvement in bloating symptoms for the patients, and synbiotics showed no significant relief compared to placebo (SMD = −0.71, 95%CI, −3.65 to 2.22) [88,90]. In addition, three studies reported an improvement in the quality of life in patients with IBS, and the results indicated no statistically significant difference between the synbiotic and placebo groups (SMD = −0.13, 95%CI, −1.01 to 0.76) [88,90,93].

Among the synbiotic-related trials included in this study, 28 cases (11.29%) of adverse events were reported in the treatment group of 248 patients, while 29 cases (11.89%) of adverse events were reported in the placebo control group of 244 patients. No serious adverse events were reported in any of the trials.

### 3.4. Efficacy and Safety of Fecal Microbiota Transplantation

A total of six double-blind RCTs related to FMT treatment were included, involving 388 individuals (246 in the intervention group) [94,95,96,97,98,99]. One trial was assessed as having a high risk of bias [94], while the remaining 5 trials were assessed as having a low risk of bias.

In comparison to placebo, FMT can effectively improve overall symptoms in patients with IBS according to the results of five RCTs involving 343 patients (SMD = −1.15, 95%CI, −1.55 to −0.75) [94,95,96,97,99]. Regarding individual symptoms in patients with IBS, only two studies reported abdominal pain post-intervention scores in patients with IBS [94,98]. One trial showed that the abdominal pain score in the FMT group was significantly lower than that in the placebo group (SMD = −0.87, 95%CI, −1.20 to −0.53) [94]. However, due to significant heterogeneity between the two studies, the meta-analysis results suggested no statistically significant effect of FMT in relieving abdominal pain (SMD = −0.26, 95%CI, −8.30 to 7.79). Only one study reported the effect of FMT on relieving bloating, evaluating the bloating symptoms of 164 patients with IBS using a continuous scale, with 109 patients in the intervention group and 55 patients in the control group [94]. The results showed that FMT significantly improved bloating symptoms compared to the placebo group. In addition, two studies reported the quality of life scores in patients with IBS, and there was no significant difference between the FMT and placebo groups in improving the quality of life in patients with IBS (SMD = 0.25, 95%CI, −13.66 to 14.15), with high heterogeneity (*I*^2^ = 93.3%, *p* = 0.0001) [94,96].

Two studies reported adverse events, with a total of 7 cases of adverse events reported in the FMT intervention group (involving 65 patients) and 10 cases of adverse events reported in the placebo control group (involving 38 patients) [97,99]. No serious adverse events were reported in either group.

### 3.5. Network Meta-Analysis of the Effectiveness of Probiotics, Prebiotics, Synbiotics, and Fecal Microbiota Transplantation

**Figure 7:**
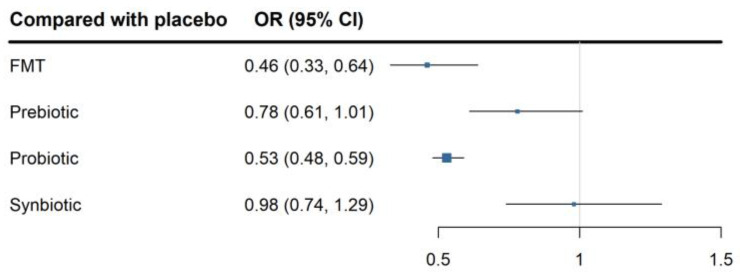
Forest plot of randomised controlled trials of different treatments versus placebo in irritable bowel syndrome: effect on global symptom change from baseline.

To further compare the effectiveness of the four treatment methods, we extracted the direct comparison data between these four treatment methods and placebo for network meta-analysis. In this network meta-analysis, a total of 36 studies on probiotics, 6 studies on prebiotics, 5 studies on synbiotics, and 4 studies on FMT were included. Using the worsening of overall symptoms as the outcome, probiotics showed the best treatment effect compared to placebo (OR = 0.53, 95%CI, 0.48 to 0.59), followed by FMT (OR = 0.46, 95%CI, 0.33 to 0.64), while prebiotics (OR = 0.78, 95%CI, 0.61 to 1.01) and synbiotics (OR = 0.98, 95%CI, 0.74 to 1.29) showed no significant difference compared to placebo (Figure 7). As shown in Table 1, probiotics had a significantly better improvement in the overall symptoms of IBS compared to prebiotics (OR = 0.68, 95%CI, 0.51 to 0.89) and synbiotics (OR = 0.54, 95%CI, 0.40 to 0.72) with statistical significance (*p* < 0.05). Similarly, FMT showed a significantly better improvement compared to prebiotics (OR = 0.62, 95%CI, 0.41 to 0.92) and synbiotics (OR = 0.60, 95%CI, 0.45 to 0.81) with statistical significance (*p* < 0.05). However, there was no significant difference between probiotics and FMT. Additionally, for the individual symptoms of IBS, we also conducted a network meta-analysis to compare the effectiveness of probiotics, prebiotics, synbiotics, and FMT. Probiotics showed a better improvement in the symptoms of abdominal pain and bloating compared to placebo. However, there was no significant difference among the four treatment methods compared to placebo in terms of improving the quality of life.

## 4. Discussion

The occurrence and development of IBS are closely related to the gut microbiota. Therefore, several strategies have been proposed to modulate the gut microbiota in IBS, including prebiotics, probiotics, dietary adjustments, and FMT [100]. Prebiotics, probiotics, synbiotics, and FMT seem to have beneficial effects on IBS, but no definitive conclusions have been drawn regarding their effectiveness [97,101,102]. It is still unclear which specific prebiotics or probiotics have superior therapeutic effects, and there is no clear consensus on whether prebiotics, probiotics, synbiotics, or FMT is the optimal choice for treatment [33,103,104]. Therefore, we conducted a systematic review and network meta-analysis to explore the methods with superior efficacy for IBS management.

This systematic review and network meta-analysis employed rigorous and reproducible methods, with two authors independently conducting literature evaluation and data extraction. We finally screened and obtained 127 articles, including 6732 participants (3707 in the intervention group), and carefully read the full text of each article to obtain the effect size data of interest. We analyzed the obtained data using a random effects model to avoid overestimating its effectiveness. Additionally, we compared the effectiveness of probiotics, prebiotics, synbiotics, and FMT in improving IBS symptoms through Bayesian network meta-analysis.

Probiotic intervention is currently the most common and extensively studied management method for IBS, with the highest number of RCTs on probiotic intervention included in this study. However, the effectiveness of probiotics has not been fully validated, and there is still controversy among different management guidelines [105]. Additionally, it is currently uncertain which specific probiotic strains or combinations of probiotics have definitive and superior efficacy [33]. The meta-analysis results of 54 RCTs on probiotics suggest that probiotics can effectively alleviate the overall symptoms of IBS. We further analyzed the overall symptom relief effects of various combinations of probiotics and individual probiotic strains (including *Bifidobacterium*, *Lactobacillus*, *Saccharomyces*, *Bacillus*, *Clostridium*, and *Escherichia. coli*) on IBS. Our meta-analysis results suggested that, among individual probiotic strains, *Bacillus* has the most optimal improvement effect on the overall symptoms of IBS, followed by *Lactobacillus* and *Saccharomyces*. However, there are limited RCT studies on these two individual strains, and the evidence is not sufficient, requiring further exploration of their effectiveness through more relevant RCT studies. *Bifidobacterium* and *Lactobacillus*, on the other hand, have relatively superior improvement effects on IBS among individual probiotic strains. The included studies on *Bifidobacterium* include *Bifidobacterium bifidum* MIMBb75, *Bifidobacterium animalis* subsp. *Lactis* UABla-12, and *Bifidobacterium infantis* 35624. *Lactobacillus* strains include *Lactobacillus acidophilus* DDS-1, *Lactobacillus casei* Shirota, *Lactobacillus acidophilus* NCFM (ATCC 700396), *Lactobacillus acidophilus*-SDC, *Lactiplantibacillus plantarum* APsulloc 331261 (GTB1), *Lactobacillus plantarum* 299V, and *Lactobacillus rhamnosus* Lcr35. These strains are promising for the treatment of IBS and have good clinical application prospects. Our meta-analysis results also suggested that the combinations of multiple probiotic strains have superior improvement effects compared to the individual probiotic strains. The probiotic combinations researched in RCTs mainly involve *Bifidobacterium*, *Lactobacillus*, *Enterococcus faecium*, *Streptococcus*, and *Pediococcus acidilactici*. These strains are potentially effective probiotics for improving IBS symptoms and provide a reference for exploring probiotic combination approaches with superior improvement effects for IBS.

The clinical symptoms of IBS mainly include bloating, abdominal pain, diarrhea, and constipation. Different subtypes of IBS have different characteristics of the gut microbiota, and their related features are related to the pathogenesis of IBS [106]. Therefore, different probiotic treatments may have inconsistent effects on different clinical symptoms of IBS. We conducted a subgroup analysis on the improvement of abdominal pain and bloating after the probiotic intervention. Our study results suggested that *Bifidobacterium* and *Lactobacillus* have a better improvement effect on abdominal pain. Probiotic combination interventions also have some good improvement effects, similar to the effect of *Bifidobacterium* single-strain intervention. For bloating, *Bifidobacterium* has the best relief effect, while the improvement effect of *Lactobacillus* single-strain and probiotic combination interventions is similar. IBS-QoL score is an important indicator for evaluating the overall improvement of IBS symptoms [78]. We also conducted subgroup analysis on IBS-QoL, and the improvement effect of probiotic combinations seems to be better than that of single strains. However, since there are few RCT studies on single-strain intervention that include IBS-QoL, more RCT studies are needed to clarify the improvement effect of single-strain intervention on the IBS-QoL score. In summary, there are few relevant studies on the improvement effect of individual strains on abdominal pain, bloating, and IBS-QoL score, and there is large heterogeneity, so no clear conclusion can be drawn. Therefore, further clinical studies are still needed to increase the evidence for their effectiveness.

The results of our study are similar to previous research findings [33,104] that probiotics are an effective method for improving IBS. Specifically, our results showed that *Bifidobacterium* and *Lactobacillus* have better relief effects on IBS. Other studies also indicated that *Bifidobacterium* is considered to be an effective strain [104,107]. However, a network meta-analysis has shown that *Bacillus coagulans* has prominent efficacy in treating patients with IBS [105]. Another study showed that mixtures of the probiotics *Bacillus coagulans* Unique IS2 or *Lactobacillus* casei Shirota have no significant effect [107]. More and more research indicates the benefits of probiotics for IBS, but which species and strains are most effective still needs further research and exploration.

Our study results suggested that both individual probiotics and probiotic combinations have an improvement effect on IBS and have certain clinical prospects. But, their mechanisms of action are not fully understood. Studies have shown that *Bifidobacterium* and *Lactobacillus* can alleviate visceral hypersensitivity in IBS mice, inhibit inflammatory factors (IL-6 and IL-17), and promote the expression of tight junction proteins (claudin-1 and occludin) [108]. *Bifidobacterium infantis* 35624 can reduce the inflammation levels of patients with IBS and has immunomodulatory effects, thereby improving discomfort such as abdominal pain, bloating, and difficult defecation [26]. Although, *Saccharomyces boulardii* CNCM I-745 can improve gastrointestinal motility and anxiety-like behavior in IBS mice by regulating gut microbiota and metabolites and affecting pain-related pathways [109]. However, these mechanisms of action cannot fully explain all the effects of probiotics in improving IBS. Therefore, it is necessary to further explore and screen probiotic strains that can significantly improve IBS through clinical studies, select specific beneficial strains for different IBS subtypes and symptoms, and combine genomics, transcriptomics, and other technologies to further elucidate their molecular mechanisms.

There is still insufficient evidence for the effectiveness of prebiotics and synbiotics in the treatment of IBS [104]. The results of this meta-analysis suggested that both prebiotics and synbiotics have no significant improvement effect on IBS, while FMT has a better improvement effect than placebo. The network meta-analysis results of the four methods suggested that probiotics and FMT have better improvement effects, while prebiotics and synbiotics have no significant improvement effects compared to placebo. The result is consistent with other research findings [110]. FMT can improve the symptoms of patients with IBS and no serious adverse events have been reported. FMT has the characteristics of being effective, easy to perform, and relatively inexpensive, and thus is a promising method for the treatment of IBS [111]. However, a study indicated that there is not enough evidence to support or refute the use of FMT for treating IBS. Considering heterogeneity between the gut microbiota of donors, not all the fecal microbiota from healthy donors is effective for all the subtypes of IBS, and whether successful colonization can be achieved is a key influencing factor for the success of FMT [112]. Therefore, different effective healthy donor gut microbiota is needed according to different gut microbiota characteristics in each subtype [106]. Therefore, based on more and larger-scale RCT studies targeting different subtypes of IBS, it is helpful to explore effective healthy donor gut microbiota for different subtypes and provide more evidence for the effective treatment of IBS by FMT.

However, there are some limitations in this article. First, heterogeneity among the literature exists in our meta-analysis. According to our analysis, this may be mainly due to the following reasons: a small sample size in some studies, different intervention measures, and different distributions of IBS types in the sample. Second, limited by the amount of included research in the field of probiotics, synbiotics and FMT, subgroup analysis by the methods of outcome assessment was not possible. Finally, the presence of unclear and high bias risk in the included articles reduces the reliability of the evidence from the data [113].

## 5. Conclusions

In summary, the results of this meta-analysis suggested that prebiotics and synbiotics do not significantly improve IBS symptoms. Among probiotics, *Bifidobacterium* and *Lactobacillus* have beneficial effects on IBS, showing a significant correlation with symptom improvement. Probiotic combinations have significant alleviating effects on IBS symptoms, abdominal pain, and IBS-QoL scores, with *Bifidobacterium* and *Lactobacillus* being the dominant strains in effective probiotic combinations. Network meta-analysis results indicated that FMT has a more favorable trend in improving IBS symptoms compared to probiotics, and it is a promising treatment method with higher safety. However, there are limited clinical studies on FMT, and more research is needed to further select effective fecal microbiota donors targeting different IBS subtypes in order to improve the efficacy of FMT.

## Figures and Tables

**Table 1:** Summary treatment effects from the network meta-analysis for failure to achieve an improvement in global IBS symptoms.

Probiotic				
0.88 (0.64, 1.19)	FMT			
0.68 (0.51, 0.89) *	0.77 (0.52, 1.14)	Prebiotic		
0.54 (0.40, 0.72) *	0.62 (0.41, 0.92) *	0.80 (0.55, 1.16)	Synbiotic	
0.53 (0.48, 0.59) *	0.60 (0.45, 0.81) *	0.78 (0.61, 1.01)	0.98 (0.74, 1.29)	Placebo

Odds ratio with 95% confidence intervals in parentheses. Comparisons, column versus row, should be read from left to right, and are ordered relative to their overall efficacy. The intervention in the top left position is ranked as best after the network meta-analysis of the direct and indirect effects. * means a statistically significance at 0.05 level.

## Data Availability

The data underlying this article are included in this published article. Data can be shared upon reasonable request to the corresponding author.

## Supplementary Materials

The following supporting information can be downloaded at: https://www.mdpi.com/article/10.3390/nu16132114/s1, Supplementary Methods (Search Strategy). Table S1: Basic characteristics of the included RCTs of probiotics versus placebo in irritable bowel syndrome; Figure S1: Funnel plot of the meta-analysis of published studies of RCTs of probiotics versus placebo in irritable bowel syndrome: effect on global symptom change from baseline; Figure S2: Risk of bias assessment summary for 54 included RCTs of probiotics versus placebo in irritable bowel syndrome: effect on global symptom change from baseline using the Cochrane Collaboration’s risk of bias tool; Figure S3: Traffic light plot of study-by-study bias assessment for 54 included RCTs of probiotics versus placebo in irritable bowel syndrome: effect on global symptom change from baseline using the Cochrane Collaboration's risk of bias tool; Figure S4: Risk of bias assessment summary for 7 included RCTs of prebiotics versus placebo in irritable bowel syndrome: effect on global symptom change from baseline using the Cochrane Collaboration's risk of bias tool; Figure S5: Traffic light plot of study-by-study bias assessment for 7 included RCTs of prebiotics versus placebo in irritable bowel syndrome: effect on global symptom change from baseline using the Cochrane Collaboration's risk of bias tool; Figure S6: Risk of bias assessment summary for 7 included RCTs of synbiotics versus placebo in irritable bowel syndrome: effect on global symptom change from baseline using the Cochrane Collaboration's risk of bias tool; Figure S7: Traffic light plot of study-by-study bias assessment for 7 included RCTs of synbiotics versus placebo in irritable bowel syndrome: effect on global symptom change from baseline using the Cochrane Collaboration's risk of bias tool; Figure S8: Risk of bias assessment summary for 6 included RCTs of FMT versus placebo in irritable bowel syndrome: effect on global symptom change from baseline using the Cochrane Collaboration's risk of bias tool; Figure S9: Traffic light plot of study-by-study bias assessment for 6 included RCTs of FMT versus placebo in irritable bowel syndrome: effect on global symptom change from baseline using the Cochrane Collaboration's risk of bias tool.

## Abbreviations

CI	Confidence intervals
ENS	Enteric nervous system
FMT	Fecal microbiota transplantation
IBS	Irritable bowel syndrome
IBS-D	Diarrhea-predominant irritable bowel syndrome
IBS-QoL	IBS quality of life
IBS-SSS	IBS symptom severity scale
OR	Odds ratio
RCT	Randomized controlled trial
RR	Relative risk
SD	Standard deviation
SMD	Standardized mean difference
