# Supplementary material for: The Efficacy of Probiotics, Prebiotics, Synbiotics, and Fecal Microbiota Transplantation in Irritable Bowel Syndrome: A Systematic Review and Network Meta-Analysis
Source: Nutrients. 2024 Jul 2;16(13):2114. doi: 10.3390/nu16132114 (PMC11243554; doi:10.3390/nu16132114)
Supplement: Supplementary file 1 [file nutrients-16-02114-s001.zip › nutrients-3050966-supplementary.pdf]

Supplementary Methods (Search Strategy)

#1 "irritable bowel syndrome"[Mesh] OR IBS OR mucous coliti* OR spastic colon OR irritable colon OR functional bowel disease* OR irritable, irritable bowel OR irritable bowel disease OR fgids OR Irritable Bowel Syndromes OR Syndrome*, Irritable Bowel OR Colon, Irritable OR Irritable Colon OR Colitis, Mucous OR Colitides, Mucous OR Mucous Colitides

#2 "probiotics"[Mesh] OR Saccharomyce* OR Bifidobacteri* OR Lactobacill* OR Streptococc* OR Enterococc* OR Lactococc* OR Bacillus bacterium OR Escherichia coli OR Bacillus OR Clostridium OR Enterococcus faecalis OR Propionibacterium

#3 "prebiotics"[Mesh] OR prebiotic OR inulin OR galactose oligosaccharide OR *fructan* OR fructooligosaccharide* OR fructo-oligosaccharide* OR oligofructose OR galactooligosaccharide* OR galacto-oligosaccharide* OR oligosaccharide* OR Idolax OR Raftilose P95

#4 "synbiotics" [Mesh] OR synbioti*

#5 "Fecal Microbiota Transplantation"[Mesh] OR Fecal Microbio* Transplant* OR Microbio* Transplant*, Fecal OR Transplant*, Fecal Microbio* OR Fecal Transplant* OR Transplant*, Fecal OR Fecal Microbio* Transfer* OR Microbio* Transfer*, Fecal OR Transfer*, Fecal Microbio* OR FMT OR stool transplant* OR Donor Feces Infusion* OR Feces Infusion*, Donor OR Infusion*, Donor Feces OR Intestinal Microbio* Transplant* OR Microbio* Transplant*, Intestinal OR Transplant*, Intestinal Microbio* OR Intestinal Microbio* Transfer* OR Microbio* Transfer*, Intestinal OR Transfer*, Intestinal Microbio* OR Intestinal Microbio* Transplant* OR Microbio* Transplant*, Intestinal OR Transplant*, Intestinal Microbio*

#6 #1 AND #2

#7 #1 AND #3

#8 #1 AND #4

#9 #1 AND #5

#10 (Randomized controlled trial OR controlled clinical trial OR random allocation OR drug therapy OR double-blind OR single-blind OR placebo OR randomly OR randomized OR clinical trial* OR trial* OR RCT OR groups OR random*)

#11 animals[Mesh] NOT humans[Mesh]

#14 #10 NOT #11

#15 #6 AND #14

#16 #7 AND #14

#17 #8 AND #14

#18 #9 AND #14

Supplementary Table S1 Basic characteristics of the included RCTs of probiotics versus placebo in irritable bowel syndrome

**Author (year)**	**Country, study design**	**Diagnosis criteria; Subtypes**	**Sample size (%female)**	**Intervention group size**	**Placebo group size**	**Age(Intervention group, mean(sd))**	**Age(Placebo group, mean(sd))**	**BMI(Intervention group, mean(sd))**	**BMI(Placebo group, mean(sd))**	**The probioics and their components**	**Dose and duration**
Guglielmetti (2011)	Germany, RCT, ITT analysis	Rome III, 21.31% IBS-D, 19.67% IBS-C, 58.2% IBS-M, 0% IBS-U	122(67.21)	60	62	36.65(12.42)	40.98(12.8)	not reported	not reported	*Bifidobacterium; Bifidobacterium bifidum* MIMBb75	QD, 1×10^9 CFU/day, 28 day
Martoni (2020)	India, RCT, ITT analysis	Rome IV, 17.27% IBS-D, 22.27% IBS-C, 0% IBS-M, 60.45% IBS-U	169(52.07)	113	56	39.41(11.8)	37.61(10.12)	not reported	not reported	*Lactobacillus; Lactobacillus acidophilus* DDS-1	QD, 1×10^10 CFU/day, 42 day
Martoni (2020)	India, RCT, ITT analysis	Rome IV, 15.98% IBS-D, 21.46% IBS-C, 0% IBS-M, 62.56% IBS-U	167(48.5)	111	56	41.6(11.11)	37.61(10.12)	not reported	not reported	*Bifidobacterium; Bifidobacterium animalis subsp. lactis* UABla-12	QD, 1×10^10 CFU/day, 42 day
Andresen (2020)	Germany, RCT, ITT analysis	Rome III, 39.95% IBS-D, 24.15% IBS-C, 7.67% IBS-M, 28.22% IBS-U	443(69.3)	221	222	40.1(12.8)	42.6(13.8)	24.5(5.3)	24.7(5)	*Bifidobacterium; Bifidobacterium bifidum* MIMBb75 (SYN-HI-001)	QD, 1×10^9 CFU/day, 56 day
Kajander (2008)	Finland, RCT, ITT analysis	Rome II, 45.35% IBS-D, 30.23% IBS-C, 24.42% IBS-M, 0% IBS-U	86(93.02)	43	43	50(13)	46(13)	25.5(3.4)	26.8(5.4)	Combination; *Lactobacillus rhamnosus* GG, L. *rhamnosus* Lc705, *Propionibacterium freudenreichii ssp. shermanii* JS and *Bifidobacterium animalis ssp. lactis* Bb12	QD, 6×10^9 CFU/day, 140 day
Sisson (2014)	UK, RCT, ITT analysis	Rome III, 37.63% IBS-D, 21.51% IBS-C, 35.48% IBS-M, 5.38% IBS-U	186(69.35)	124	62	39.1(10.5)	36.8(10.8)	not reported	not reported	Combination; *Lactobacillus rhamnosus* NCIMB 30174, *Lactobacillus plantarum* NCIMB 30173, *Lactobacillus*	QD, 1.2×10^1 0 CFU/day, 84 day

Supplementary Table S1 (Continued)

Author (year)	Country, study design	Diagnosis criteria; Subtypes	Sample size (%female)	Intervention group size	Placebo group size	Age(Intervention group, mean(sd))	Age(Placebo group, mean(sd))	BMI(Intervention group, mean(sd))	BMI(Placebo group, mean(sd))	The probioics and their components	Dose and duration
										acidophilus NCIMB 30175 and *Enterococcus faecium* NCIMB 30176	
Urgesi (2014)	Italy, RCT, not reported	Rome III, subtype not reported	52(65.38)	26	26	42(15.9)	36(12.1)	not reported	not reported	*Bacillus; Bacillus coagulans* (Colinox ®)	TID, not reported, 28 day
Kim (2003)	USA, RCT, ITT analysis	Rome II, 100% IBS-D, 0% IBS-C, 0% IBS-M, 0% IBS-U	25(72)	12	13	48(19.75)	38(12.26)	not reported	not reported	*Bifidobacterium* Combination; VSL#3 three strains of *Bifidobacterium (B. longum, B. infantis and B. breve*); four strains of *Lactobacillus (L. acidophilus, L. casei, L. bulgaricus and L. plantarum*); and one strain of *Streptococcus* (*S. salivarius subspecies thermophilus*)	BID, 4.5×10^11 CFU/day, 56 day
Helo (2019)	Jordan, RCT, not reported	Rome III, 17.29% IBS-D, 65.99% IBS-C, 17.58% IBS-M, 0% IBS-U	not reported	177	170	not reported	not reported	not reported	not reported	*Cerevisiae; Cerevisiae* CNCM I-3856	BID, not reported, 28 day
Majeed (2018)	India, RCT, not reported	Rome III, subtype not reported	40(85)	20	20	40.36(10.28)	43.88(9.85)	not reported	not reported	*Bacillus; Bacillus coagulans* MTCC 5856	QD, 2×10^9 CFU/day, 90 day
Spiller (2016)	France, RCT, ITT analysis	Rome III, 20.84% IBS-D, 47.49% IBS-C, 31.66% IBS-M, 0% IBS-U	379(83.64)	192	187	45.3(15.7)	45.4(14.1)	not reported	not reported	*Saccharomyces; Saccharomyces cerevisiae* I-3856	QD, 8×10^9 CFU/day, 84 day

Supplementary Table S1 (Continued)

Author (year)	Country, study design	Diagnosis criteria; Subtypes	Sample size (%female)	Intervention group size	Placebo group size	Age(Intervention group, mean(sd))	Age(Placebo group, mean(sd))	BMI(Intervention group, mean(sd))	BMI(Placebo group, mean(sd))	The probioics and their components	Dose and duration
Hong (2009)	Korea, RCT, ITT analysis	Rome III, 45.71% IBS-D, 20% IBS-C, 8.57% IBS-M, 25.71% IBS-U	70(32.86)	36	34	36(12)	38(17.49)	not reported	not reported	Combination; *Bifidobacterium bifidum* BGN4;*Bifidobacterium lactis* AD011; *Lactobacillus acidophilus* AD031;and *Lactobacillus casei* IBS041	BID, 4×10^10 CFU/day, 56 day
Cui (2012)	China, RCT, not reported	Rome III, 48.33% IBS-D, 30% IBS-C, 11.67% IBS-M, 10% IBS-U	60(70)	37	23	42.92(15.09)	47.45(15.36)	22.08(3.23)	20.77(2.65)	Combination; *Bifidobacterium longum* DSM 20219 and *Lactobacillus acidophilus* DSM 20079	TID, not reported, 28 day
Preston (2018)	USA, RCT, ITT analysis	Rome III, subtype not reported	113(60.18)	75	38	39.9(14)	40.6(13.4)	not reported	not reported	Combination; *Lactobacillus acidophilus* CL1285, *Lactobacillus casei* LBC80R and *Lactobacillus rhamnosus* CLR2	QD, 1×10^11 CFU/day, not reported
Bai (2022)	China, RCT, ITT analysis	Rome IV, subtype not reported	290(44.83)	145	145	42.41(11.92)	45.74(12.4)	23.1(3.14)	23.69(2.75)	Combination; *Bifidobacterium infantis, Lactobacillus acidophilus, Enterococcus faecalis*,plus GMCC 0460.4	TID, 1.65×10^6 CFU/day, 28 day
Thijssen (2016)	Holland, RCT, ITT analysis	Rome II, 30% IBS-D, 25% IBS-C, 28.75% IBS-M, 16.25% IBS-U	80(68.75)	39	41	41.1(14.8)	42.4(13.5)	25.43(4.53)	24.06(3.82)	*Lactobacillus; Lactobacillus casei Shirota* (LcS)	BID, 1.3×10^10 CFU/day, 56 day
Yoon (2014)	Korea, RCT, ITT analysis	Rome III, 53.06% IBS-D, 40.82% IBS-C,	49(65.31)	25	24	45.9(13.7)	43.1(15.1)	not reported	not reported	Combination; LacClean Gold-S（*Bifidobacterium*	BID, 1×10^10 CFU/day,

Supplementary Table S1 (Continued)

Author (year)	Country, study design	Diagnosis criteria; Subtypes	Sample size (%female)	Intervention group size	Placebo group size	Age(Intervention group, mean(sd))	Age(Placebo group, mean(sd))	BMI(Intervention group, mean(sd))	BMI(Placebo group, mean(sd))	The probioics and their components	Dose and duration
		6.12% IBS-M, 0% IBS-U								*longum, Bifidobacterium bifidum, Bifidobacterium lactis, Lactobacillus acidophilus, Lactobacillus rhamnosus, and Streptococcus thermophilus*）	28 day
Ludidi (2014)	Holland, RCT, ITT analysis	Rome III, 42.5% IBS-D, 10% IBS-C, 30% IBS-M, 15% IBS-U	40(67.5)	21	19	40(10.08)	41.1(18.31)	25.8(5.04)	25.1(3.92)	Combination; *Bifidobacterium lactis* W52, *Lactobacillus casei* W56, *Lactobacillus salivarius* W57, *Lactococcus lactis* W58, *Lactobacillus acidophilus* NCFM, and *Lactobacillus rhamnosus* W71	QD, 5×10^9 CFU/day, 42 day
Abbas (2014)	Pakistan, RCT, ITT analysis	Rome III, 100% IBS-D, 0% IBS-C, 0% IBS-M, 0% IBS-U	72(26.39)	37	35	37.7(11.6)	33(12)	not reported	not reported	*Saccharomyces; Saccharomyces boulardii*	QD, not reported, 42 day
Charbonneau (2013)	Ireland, RCT, PP analysis	Rome II, subtype not reported	76(81.58)	33	28	47(12.24)	43.2(12.23)	not reported	not reported	*Bifidobacterium; Bifidobacterium infantis* 35624	QD, 1×10^9 CFU/day, 56 day
Roberts (2013)	UK, RCT, ITT analysis	Rome III, subtype not reported	179(83.24)	91	88	44.66(11.98)	43.71(12.76)	not reported	not reported	Combination; *Bifidobacterium lactis* CNCM I-2494, *S. thermophilus and L. bulgaricus*	BID/BIW, 1.37×10^10 CFU/day, 14/84 day
Choi (2011)	Korea, RCT, ITT analysis	Rome II, 58.89% IBS-D,	90(58.89)	45	45	40.2(13.1)	40.6(12.9)	not reported	not reported	*Saccharomyces; Saccharomyces*	BID, 4×10^11

Supplementary Table S1 (Continued)

Author (year)	Country, study design	Diagnosis criteria; Subtypes	Sample size (%female)	Intervention group size	Placebo group size	Age(Intervention group, mean(sd))	Age(Placebo group, mean(sd))	BMI(Intervention group, mean(sd))	BMI(Placebo group, mean(sd))	The probioics and their components	Dose and duration
		0% IBS-C, 23.33% IBS-M, 0% IBS-U								*boulardii*	CFU/day, 28 day
Michail (2011)	USA, RCT, not reported	Rome III, 100% IBS-D, 0% IBS-C, 0% IBS-M, 0% IBS-U	24(66.67)	15	9	not reported	not reported	not reported	not reported	*Bifidobacterium* Combination; VSL#3	QD, 9×10^11 CFU/day, 56 day
Williams (2009)	UK, RCT, PP analysis	Rome II, 11.54% IBS-D, 26.92% IBS-C, 61.54% IBS-M, 0% IBS-U	52(86.54)	28	24	40(12)	38(11)	not reported	not reported	Combination; *Lactobacillus acidophilus* CUL60 (NCIMB30157) and CUL21 (NCIMB 30156), *Bifidobacterium lactis* CUL34(NCIMB 30172) and *Bifidobacterium bifidum* CUL20 (NCIMB 30153)	QD, 2.5×10^1 0 CFU/day, 56 day
Enck (2008)	Germany, RCT, ITT analysis	Kruis, subtype not reported	297(49.49)	149	148	49.8(12.75)	49.4(14.5)	24.4(5.1)	24(4.03)	Combination; *Enterococcus faecalis* (DSM 16440) and *E. coli* (DSM 17252)	TID, 4.5-13.5× 10^7 CFU/day, 56 day
Zeng (2008)	China, RCT, PP analysis	Rome II, subtype not reported	29(34.48)	14	15	44.6(12.4)	45.8(9.2)	not reported	not reported	Combination; *Streptococcus thermophilus, Lactobacillus bulgaricus, Lactobacillus acidophilus* and *Bifidobacterium Longum*	BID, 5.2×10^1 0 CFU/day, 28 day
Drouault-Holowacz (2008)	France, RCT, PP analysis	Rome II, 29% IBS-D, 29% IBS-C, 41%	100(76)	48	52	47(14)	44(14)	not reported	not reported	Combination; *B. longum* LA 101, *Lb.acidophilus* LA	QD, 1×10^10 CFU/day,

Supplementary Table S1 (Continued)

Author (year)	Country, study design	Diagnosis criteria; Subtypes	Sample size (%female)	Intervention group size	Placebo group size	Age(Intervention group, mean(sd))	Age(Placebo group, mean(sd))	BMI(Intervention group, mean(sd))	BMI(Placebo group, mean(sd))	The probioics and their components	Dose and duration
		IBS-M, 1% IBS-U								102, *L. lactis* LA 103 and *S. thermophilus* LA 104	28 day
Whorwell (2006)	Ireland, RCT, ITT analysis	Rome II, subtype not reported	121(100)	90	31	41.8(10.44)	42.4(10.46)	not reported	not reported	*Bifidobacterium; Bifidobacterium infantis* 35624	QD, 1×10^10 CFU/day, 28 day
Whorwell (2006)	Ireland, RCT, ITT analysis	Rome II, 29.01% IBS-D, 9.94% IBS-C, 11.33% IBS-M, 0% IBS-U	121(100)	90	31	42.7(10.44)	42.4(10.46)	not reported	not reported	*Bifidobacterium; Bifidobacterium infantis* 35624	QD, 1×10^8 CFU/day, 28 day
Whorwell (2006)	Ireland, RCT, ITT analysis	Rome II, subtype not reported	120(100)	90	30	40.8(10.44)	42.4(10.46)	not reported	not reported	*Bifidobacterium; Bifidobacterium infantis* 35624	QD, 1×10^6 CFU/day, 28 day
Kim (2005)	USA, RCT, ITT analysis	Rome II, subtype not reported	48(93.75)	24	24	40(14.7)	46(14.7)	not reported	not reported	*Bifidobacterium* Combination; VSL#3 *Bifidobacterium* (*B. longum, B. infantis* and *B. breve*);*Lactobacillus* (*L. acidophilus, L. casei, L. delbrueckii ssp. bulgaricus and L. plantarum*); and *Streptococcus salivarius ssp. Thermophilus*	BID, 9×10^11 CFU/day, 56 day
Kajander (2005)	Finland, RCT, PP analysis	Rome II, 47.57% IBS-D, 23.3% IBS-C, 29.13% IBS-M, 0% IBS-U	103(76.7)	41	40	46(10.5)	45(11)	25.7(4.93)	24.4(5.75)	Combination; LGG, *L. rhamnosus* LC705, *B. breve* Bb99 and *P. freudenreichii ssp. shermanii* JS	QD, 8-9×10^9 CFU/day, 180 day
Pinto-Sanchez (2017)	Canada, RCT, ITT analysis	Rome III, 61.36% IBS-D,	44(54.55)	22	22	46.5(20.74)	40(22.96)	25.1(5.11)	24.6(5.33)	*Bifidobacterium; Bifidobacterium*	QD, 1×10^10

Supplementary Table S1 (Continued)

Author (year)	Country, study design	Diagnosis criteria; Subtypes	Sample size (%female)	Intervention group size	Placebo group size	Age(Intervention group, mean(sd))	Age(Placebo group, mean(sd))	BMI(Intervention group, mean(sd))	BMI(Placebo group, mean(sd))	The probioics and their components	Dose and duration
		0% IBS-C, 38.64% IBS-M, 0% IBS-U								*longum* NCC3001	CFU/day, 42 day
Lyra (2016)	Finland, RCT, ITT analysis	Rome III, 38.55% IBS-D, 17.18% IBS-C, 43.51% IBS-M, 0.76% IBS-U	197(77.16)	131	66	47.2(12.5)	49.4(12.9)	24.5(3.9)	24.9(3.7)	*Lactobacillus*; *L.acidophilus* NCFM (ATCC 700396)	QD, 1×10^10 CFU/day, 84 day
Lyra (2016)	Finland, RCT, ITT analysis	Rome III, 38.46% IBS-D, 17.31% IBS-C, 43.85% IBS-M, 0.38% IBS-U	194(72.17)	129	65	47.1(13.3)	49.4(12.9)	24.7(3.7)	24.9(3.7)	*Lactobacillus*; *L.acidophilus* NCFM (ATCC 700397)	QD, 1×10^9 CFU/day, 84 day
Gupta (2021)	India, RCT, PP analysis	Rome IV, subtype not reported	40(30)	19	19	36.2(9.81)	34.8(11.06)	23.37(3.23)	24.03(2.99)	*Bacillus*; *Bacillus coagulans* LBSC [DSM17654]	TID, 6×10^9 CFU/day, 80 day
Oh (2019)	Vietnam, RCT, PP analysis	Rome III, 42% IBS-D, 0% IBS-C, 20% IBS-M, 38% IBS-U	50(72)	26	24	32.5(9.26)	33(12.22)	22.2(2.74)	20.7(2.96)	*Lactobacillus* Combination; *Lactobacillus species*(*L. paracasei, L. salivarius, and L. plantarum*) *Foodis Lactobacillus*	QD, 1×10^9 CFU/day, 28 day
Mourey (2022)	France, RCT, ITT analysis	Rome IV, subtype not reported	456(85.96)	230	226	41.2(13.96)	39.9(14.56)	not reported	not reported	*Saccharomyces; S. cerevisiae* CNCM I-3856	QD, 8×10^9 CFU/day, 56 day
Gayathri (2019)	India, RCT, PP analysis	Rome III, 65% IBS-D, 24% IBS-C, 11% IBS-M, 0% IBS-U	100(34)	48	44	42.25(15.44)	39.604(12.79)	not reported	not reported	*Saccharomyces; Saccharomyces cerevisiae* CNCM I-3856	BID, 4×10^9 CFU/day, 56 day
Sinn (2008)	Korea, RCT, ITT analysis	Rome III, 10% IBS-D, 27.5%	40(65)	20	20	41.9(14.4)	47.5(11)	21.9(3.6)	22.4(2.2)	*Lactobacillus; Lactobacillus*	BID, 2×10^9

Supplementary Table S1 (Continued)

Author (year)	Country, study design	Diagnosis criteria; Subtypes	Sample size (%female)	Intervention group size	Placebo group size	Age(Intervention group, mean(sd))	Age(Placebo group, mean(sd))	BMI(Intervention group, mean(sd))	BMI(Placebo group, mean(sd))	The probioics and their components	Dose and duration
		IBS-C, 62.5% IBS-M, 0% IBS-U								*acidophilus*-SDC 2012, 2013	CFU/day, 28 day
Jafari (2014)	Iran, RCT, ITT analysis	Rome III, subtype not reported	108(60.19)	54	54	36.6(12.1)	36.8(11)	not reported	not reported	Combination; Probio-Tec® Quatro-cap-4(*Bifidobacterium animalis subsp.lactis*BB-12®, *Lactobacillus acidophilus* LA-5®, *Lactobacillus delbrueckii subsp. bulgaricus* LBY-27, *Streptococcus thermophilus* STY-31)	BID, 8×10^9 CFU/day, 28 day
Majeed (2016)	India, RCT, PP analysis	Rome III, subtype not reported	36(52.78)	18	17	36.2(11.07)	35.4(10.75)	24.6(3.14)	24.1(3.98)	*Bacillus; Bacillus coagulans* MTCC 5856	QD, 2×10^9 CFU/day, 90 day
Madempudi (2019)	India, RCT, PP analysis	Rome III, subtype not reported	108(27.78)	53	55	44.4(10)	42.3(9.75)	not reported	not reported	*Bacillus; B. coagulans Unique* IS2	QD, 2×10^9 CFU/day, 56 day
Mezzasalma (2016)	Italy, RCT, PP analysis	Rome III, 100% IBS-C	not reported	50	25	36(11.9)	38.1(13.5)	not reported	not reported	*Lactobacillus* Combination; *L. acidophilus* and *L. reuteri*	QD, 1×10^10 CFU/day, 60 day
Mezzasalma (2016)	Italy, RCT, PP analysis	Rome III, 100% IBS-C	not reported	50	25	38(12.1)	38.1(13.5)	not reported	not reported	Combination; *L. plantarum, L. rhamnosus*, and *B. animalis subsp. lactis*	QD, 1.5×10^10 CFU/day, 60 day
Kruis (2012)	Germany, RCT, ITT analysis	Rome II, 45% IBS-D, 29.17% IBS-C, 0% IBS-M, 0% IBS-U	120(76.67)	60	60	46.3(12.1)	45.1(12.7)	not reported	not reported	*Escherichia coli; Escherichia coli Nissle* 1917 (MUTAFLOR®)	QD, 5-50×10^10 CFU/day, 84 day
Jung (2022)	Korea, RCT, PP	Rome IV,	26(69.23)	18	8	39.84(6.44)	39.38(3.16)	24(3.44)	24.4(3.84)	Lactobacillus;	QD,

Supplementary Table S1 (Continued)

Author (year)	Country, study design	Diagnosis criteria; Subtypes	Sample size (%female)	Intervention group size	Placebo group size	Age(Intervention group, mean(sd))	Age(Placebo group, mean(sd))	BMI(Intervention group, mean(sd))	BMI(Placebo group, mean(sd))	The probioics and their components	Dose and duration
	analysis	100% IBS-D, 0% IBS-C, 0% IBS-M, 0% IBS-U								*Lactiplantibacillus plantarum APsulloc* 331261 (GTB1)	1×10^10 CFU/day, 28 day
Skrzydlo-Radomanska (2021)	Poland, RCT, PP analysis	Rome III, subtype not reported	48(64.58)	25	23	45.5(11.1)	40.7(14.4)	26.5(4.7)	25.2(4.2)	Combination; *Bifidobacterium breve* BB010;*Bifidobacterium breve* BL020;*Bifidobacterium bifidum* BF030;*Bifidobacterium lactis* BL040; *Lactobacillus rhamnosus* LR110;*Lactobacillus paracasei* LPC100; *Lactobacillus acidophilus* LA120;*Lactobacillus casei* LC130;*Lactobacillus plantarum* LP140; *Streptococcus thermophilus* ST250	BID, 5×10^9 CFU/day, 56 day
Niedzielin (2001)	Poland, RCT, ITT analysis	Manning, 2.5% IBS-D, 52.5% IBS-C, 45% IBS-M, 0% IBS-U	40(80)	20	20	45(18)	42(15)	23.3(4.5)	24.1(2.7)	*Lactobacillus; Lactobacillus plantarum* 299V	BID, 2×10^10 CFU/day, 28 day
Stevenson (2014)	South Africa, RCT, ITT analysis	Rome II, subtype not reported	81(97.53)	54	27	48.15(13.48)	47.27(12.15)	28.83(7.12)	28.88(7.74)	*Lactobacillus; Lactobacillus plantarum* 299V	QD, 1×10^10 CFU/day, 56 day
Hong (2011)	Korea, RCT, PP analysis	Rome III, 52.05% IBS-D, 8.22% IBS-C, 39.73% IBS-M, 0%	73(69.86)	37	36	33(8.5)	33(7.75)	not reported	not reported	Combination; *Lactobacillus sp.*HY7801, *Bifidobacterium longum* HY8004, and	TID, 12×10^9 CFU/day, 56 day

Supplementary Table S1 (Continued)

Author (year)	Country, study design	Diagnosis criteria; Subtypes	Sample size (%female)	Intervention group size	Placebo group size	Age(Intervention group, mean(sd))	Age(Placebo group, mean(sd))	BMI(Intervention group, mean(sd))	BMI(Placebo group, mean(sd))	The probioics and their components	Dose and duration
		IBS-U								*Lactobacillus brevis* HY7401	
Barraza-Ortiz (2021)	Mexico, RCT, ITT analysis	Rome IV, 55.56% IBS-D, 0% IBS-C, 44.44% IBS-M, 0% IBS-U	36(61.11)	18	18	45.1(9.2)	46.1(5.2)	26.8(5.2)	24.6(3.4)	Combination; I3.1（*lactic acid bacteria: L. plantarum* CECT 7484, *L. plantarum* CECT 7485, and *P. acidilactici* CECT 7483）	QD, 3×10^9 CFU/day, 42 day
Lorenzo-Zuniga (2014)	Spain, RCT, ITT analysis	Rome III, subtype not reported	43(60.47)	28	15	47.5(13.1)	46.5(13.1)	24.7(3.9)	26.4(5.2)	Combination; I3.1(*Lactobacillus plantarum* (CECT7484 and CECT7485) and one *Pediococcus acidilactici* (CECT7483)	QD, 1-3×10^10 CFU/day, 42 day
Lorenzo-Zuniga (2014)	Spain, RCT, ITT analysis	Rome III, subtype not reported	41(65.85)	27	14	46.3(11.6)	46.5(13.1)	25.6(5.1)	26.4(5.2)	Combination; I3.1(*Lactobacillus plantarum* (CECT7484 and CECT7485) and one *Pediococcus acidilactici* (CECT7483)	QD, 3-6×10^9 CFU/day, 42 day
Pineton (2015)	France, RCT, PP analysis	Rome III, 28.49% IBS-D, 46.93% IBS-C, 24.58% IBS-M, 0% IBS-U	179(86.03)	86	93	42.5(12.5)	45.4(14)	not reported	not reported	*Saccharomyces; Saccharomyces cerevisiae* CNCM I-3856	QD, 4×10^9 CFU/day, 56 day
Simren (2010)	Sweden, RCT, ITT analysis	Rome II, 35.14% IBS-D, 14.86% IBS-C, 50% IBS-M, 0% IBS-U	74(70.27)	37	37	42(15)	44(16)	not reported	not reported	Combination; *Lactobacillus paracasei, ssp. paracasei* F19, *Lactobacillus acidophilus* La5 and *Bifidobacterium lactis*	BID, 2×10^10 CFU/day, 56 day

Supplementary Table S1 (Continued)

Author (year)	Country, study design	Diagnosis criteria; Subtypes	Sample size (%female)	Intervention group size	Placebo group size	Age(Intervention group, mean(sd))	Age(Placebo group, mean(sd))	BMI(Intervention group, mean(sd))	BMI(Placebo group, mean(sd))	The probioics and their components	Dose and duration
										Bb12	
Dapoigny (2012)	France, RCT, ITT analysis	Rome III, 30% IBS-D, 22% IBS-C, 34% IBS-M, 14% IBS-U	50(70)	25	25	46.1(11.3)	48(10.8)	23.4(4.9)	24.5(4)	*Lactobacillus*; LCR35 *Lactobacillus casei variety rhamnosus*	QD, 6×10^8 CFU/day, 28 day
Sadrin (2020)	France, RCT, ITT analysis	Rome III, subtype not reported	80(71.25)	40	40	48.9(8.4)	48.9(8)	not reported	not reported	*Lactobacillus* Combination; Two *Lactobacillus acidophilus*(*L. acidophilus* NCFM ATCC SD5221+*L. acidophilus subsp.helveticus* LAFTI L10 (strain number CBS 116.411)	BID, 1×10^10 CFU/day, 56 day
Cha (2010)	Korea, RCT, ITT analysis	Rome III, 100% IBS-D, 0% IBS-C, 0% IBS-M, 0% IBS-U	50(48)	25	25	37.9(12.4)	40.3(11.2)	23.03(3.31)	22.93(2.88)	Combination; *Lactobacillus acidophilus, Lactobacillus plantarum, Lactobacillus rhamnosus, Bifidobacterium breve, Bifidobacterium lactis, Bifidobacterium longum, and Streptococcus thermophilus*	QD, 1×10^10 CFU/day, 56 day
Sun (2018)	China, RCT, PP analysis	Rome III, subtype not reported	200(42)	85	81	43(12.45)	44.91(13.01)	not reported	not reported	*Clostridium; Clostridium butyricum* (CB)	TID, 5.67×10^ 7 CFU/day, 28 day

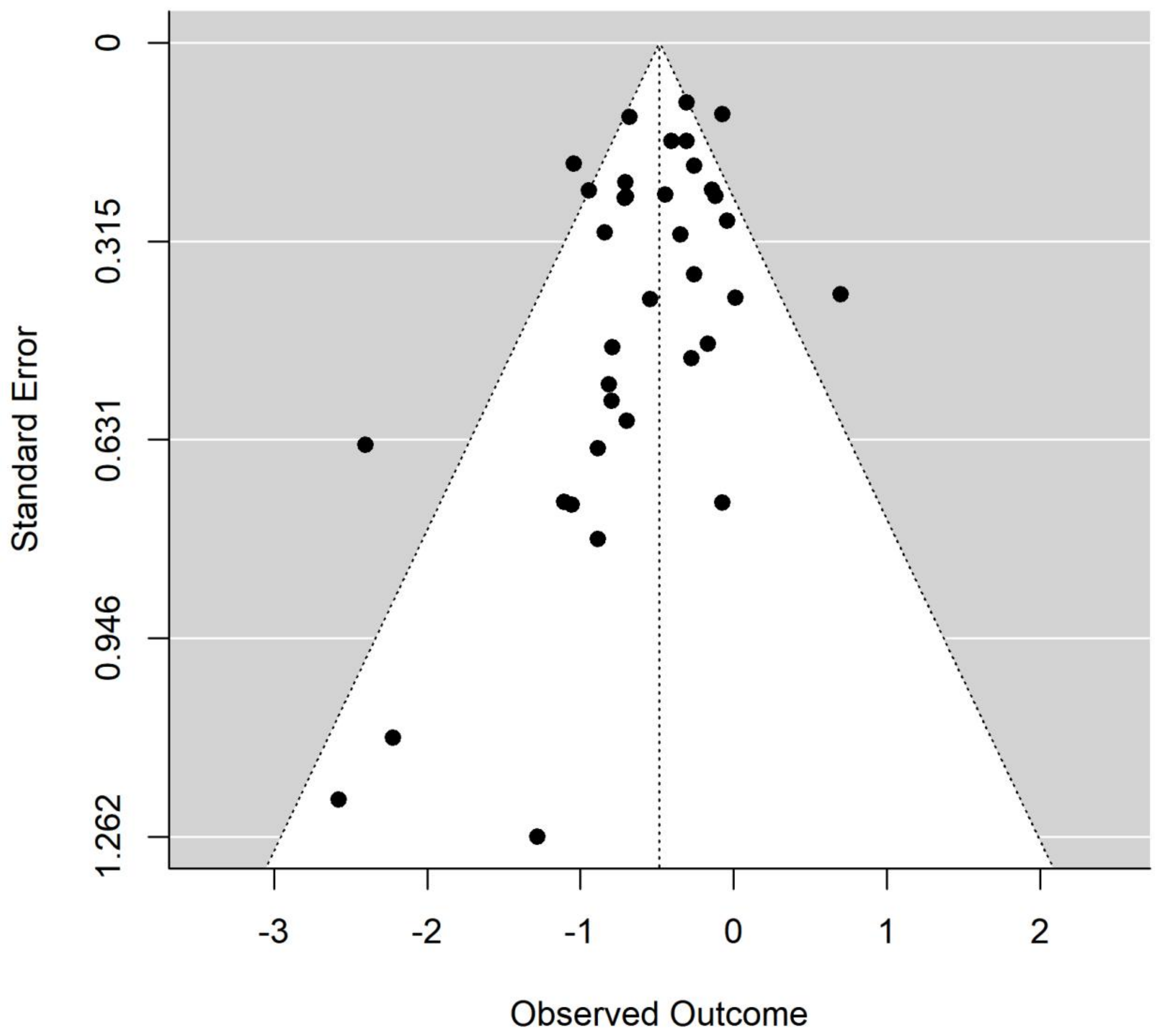

Supplementary Figure S1 Funnel plot of the meta-analysis of published studies of RCTs of probiotics versus placebo in irritable bowel syndrome: effect on global symptom change from baseline.

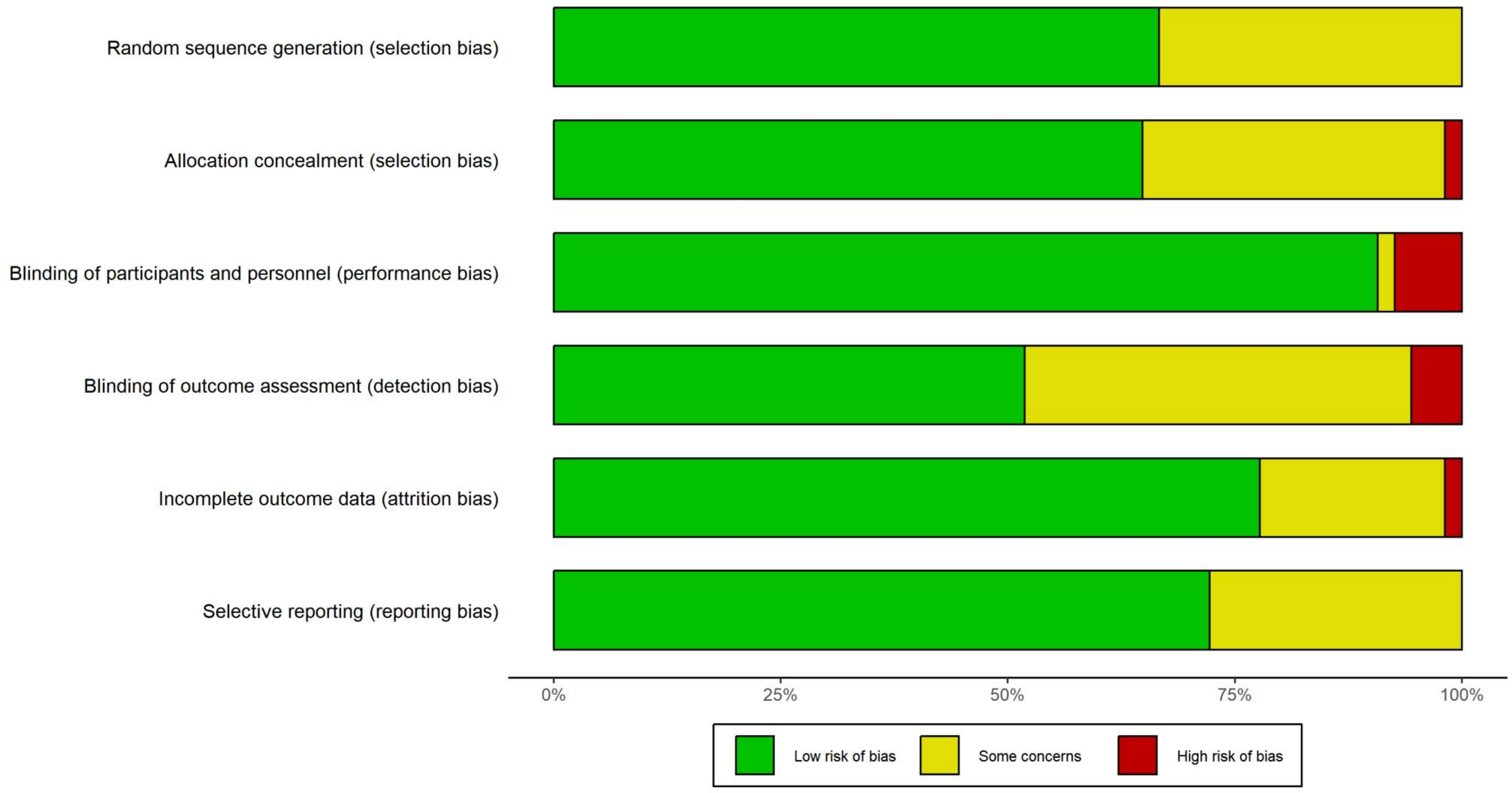

Supplementary Figure S2 Risk of bias assessment summary for 54 included RCTs of probiotics versus placebo in irritable bowel syndrome: effect on global symptom change from baseline using the Cochrane Collaboration's risk of bias tool.

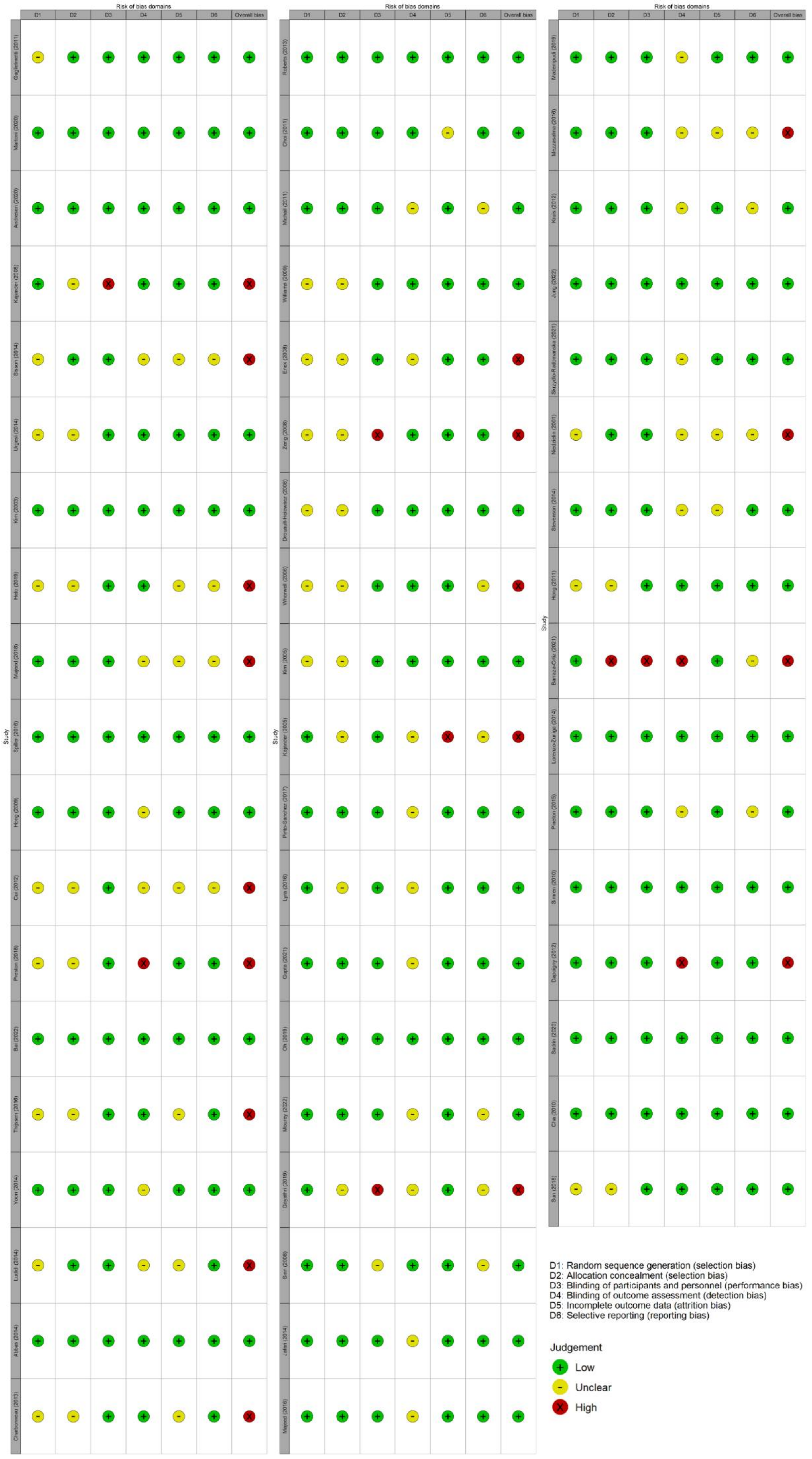

Supplementary Figure S3 Traffic light plot of study-by-study bias assessment for 54 included RCTs of probiotics versus placebo in irritable bowel syndrome: effect on global symptom change from baseline using the Cochrane Collaboration's risk of bias tool.

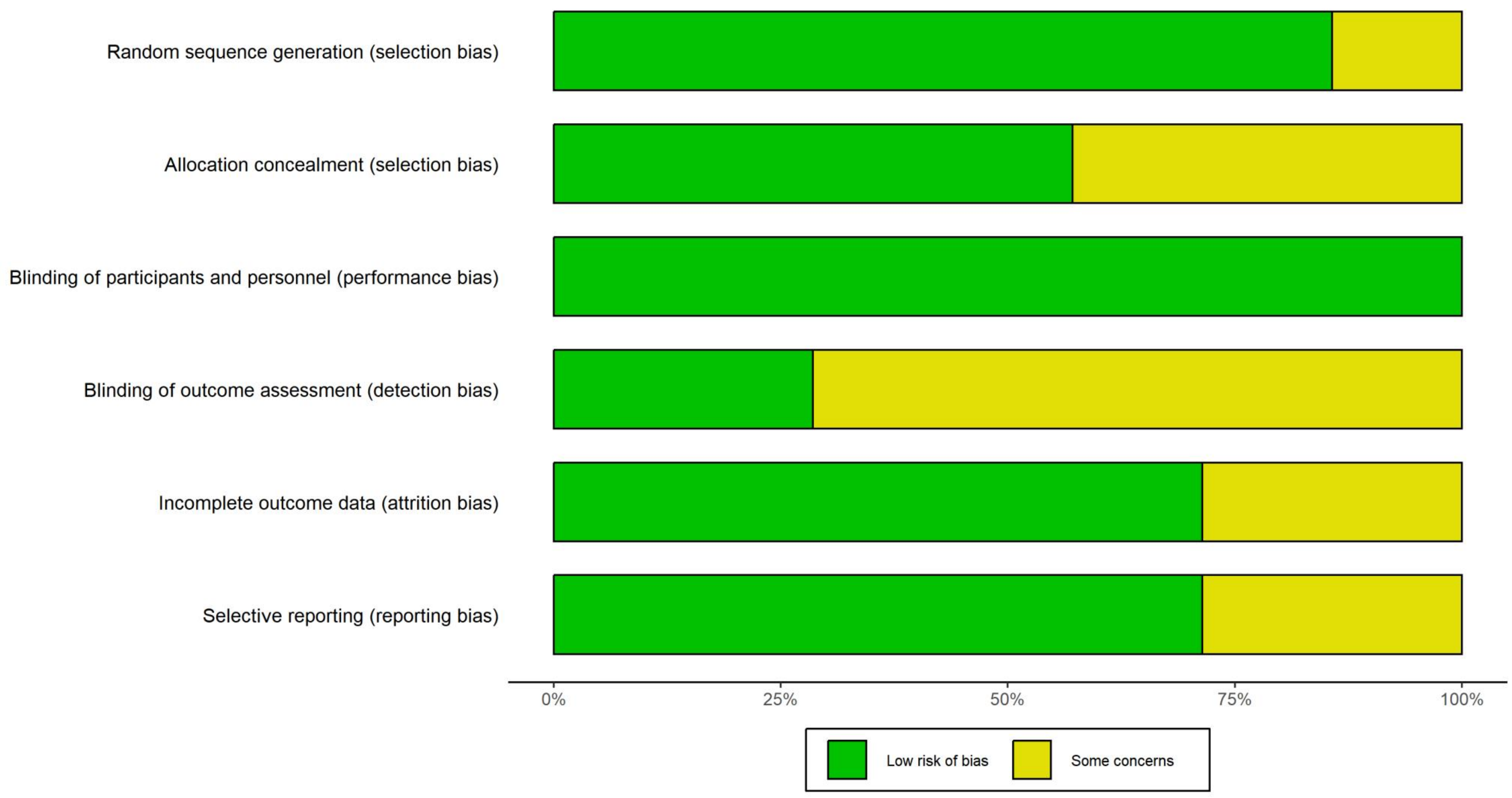

Supplementary Figure S4 Risk of bias assessment summary for 7 included RCTs of prebiotics versus placebo in irritable bowel syndrome: effect on global symptom change from baseline using the Cochrane Collaboration's risk of bias tool.

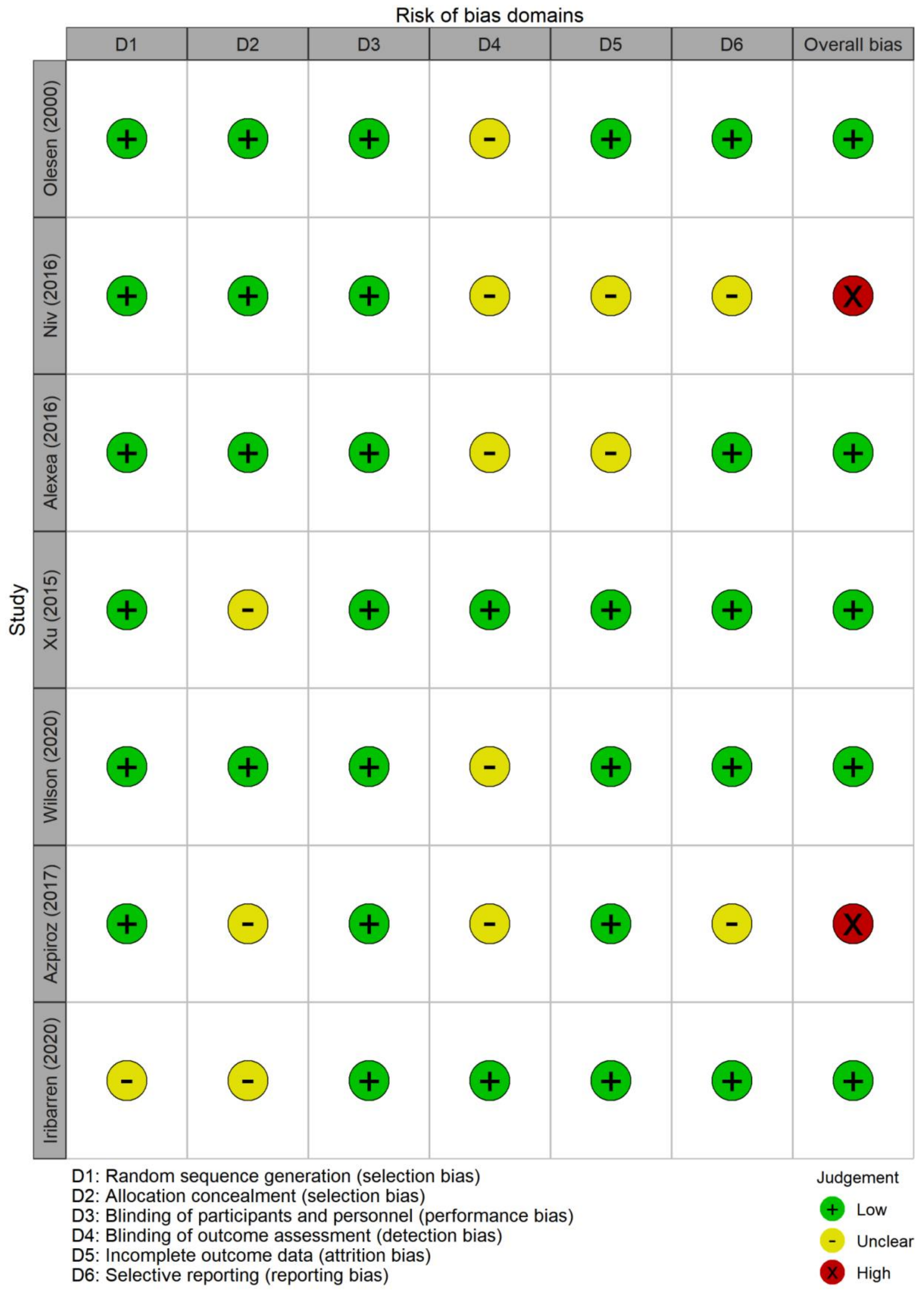

Supplementary Figure S5 Traffic light plot of study-by-study bias assessment for 7 included RCTs of prebiotics versus placebo in irritable bowel syndrome: effect on global symptom change from baseline using the Cochrane Collaboration's risk of bias tool.

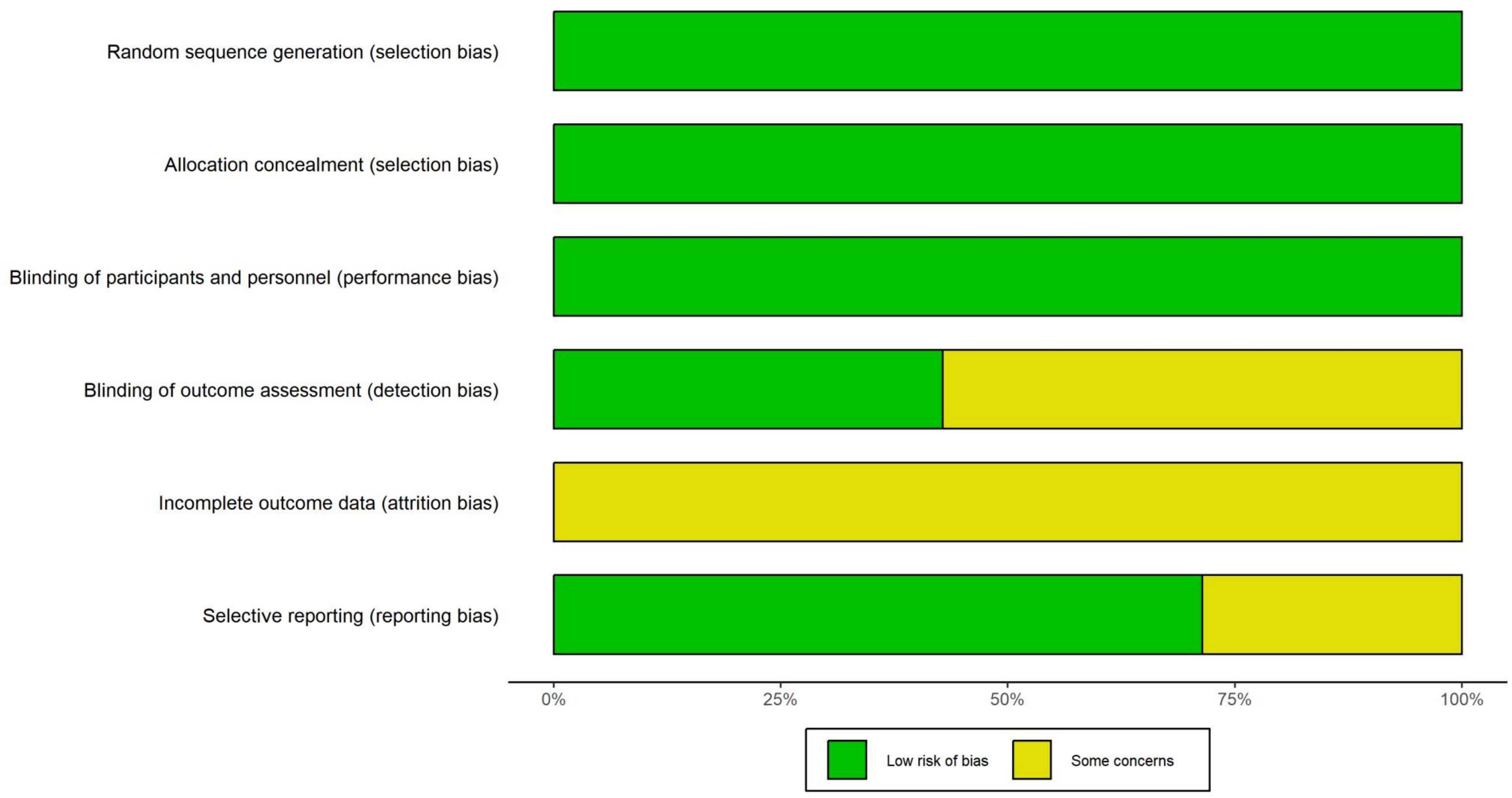

Supplementary Figure S6 Risk of bias assessment summary for 7 included RCTs of synbiotics versus placebo in irritable bowel syndrome: effect on global symptom change from baseline using the Cochrane Collaboration's risk of bias tool.

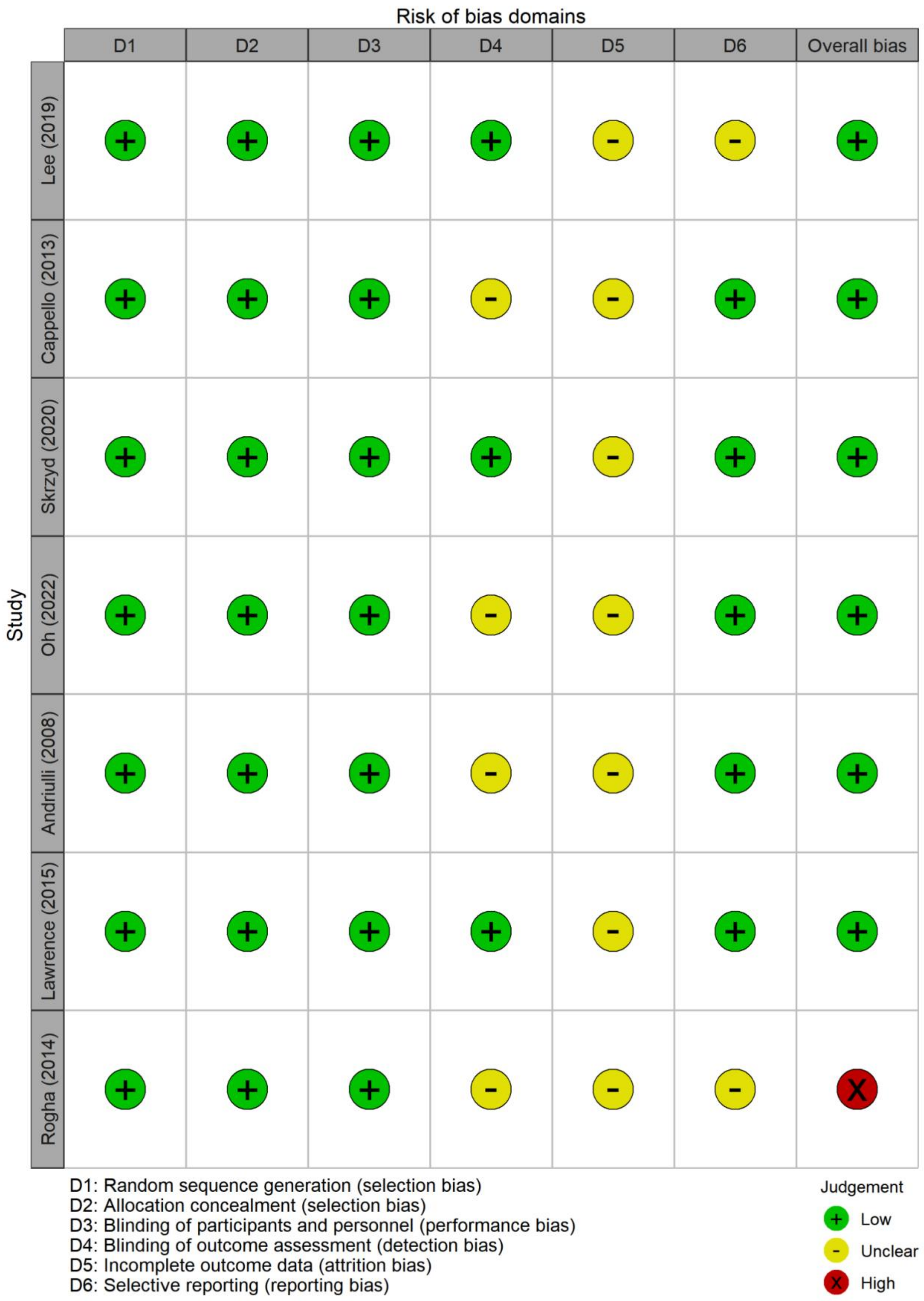

Supplementary Figure S7 Traffic light plot of study-by-study bias assessment for 7 included RCTs of synbiotics versus placebo in irritable bowel syndrome: effect on global symptom change from baseline using the Cochrane Collaboration's risk of bias tool.

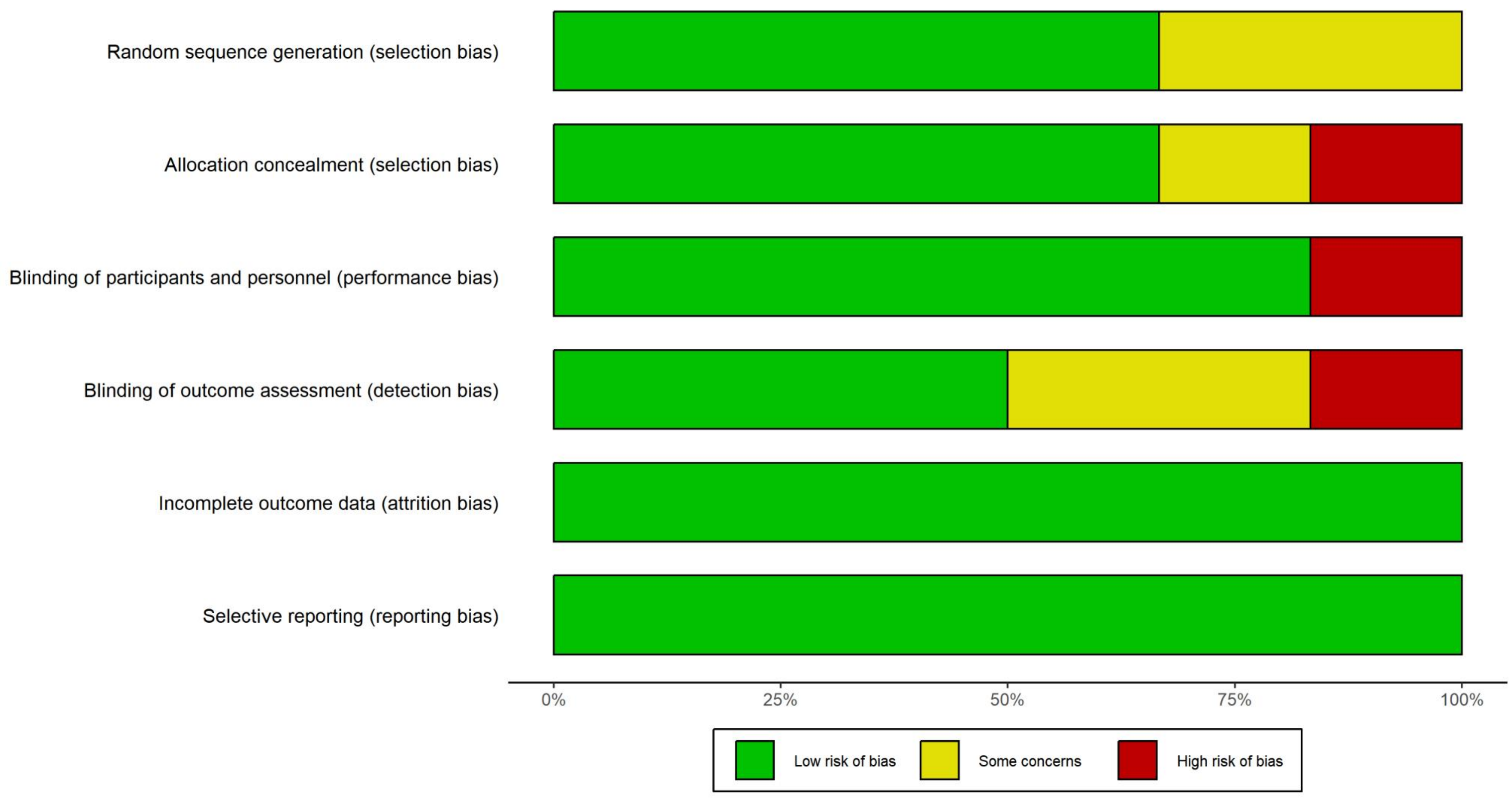

Supplementary Figure S8 Risk of bias assessment summary for 6 included RCTs of FMT versus placebo in irritable bowel syndrome: effect on global symptom change from baseline using the Cochrane Collaboration's risk of bias tool.

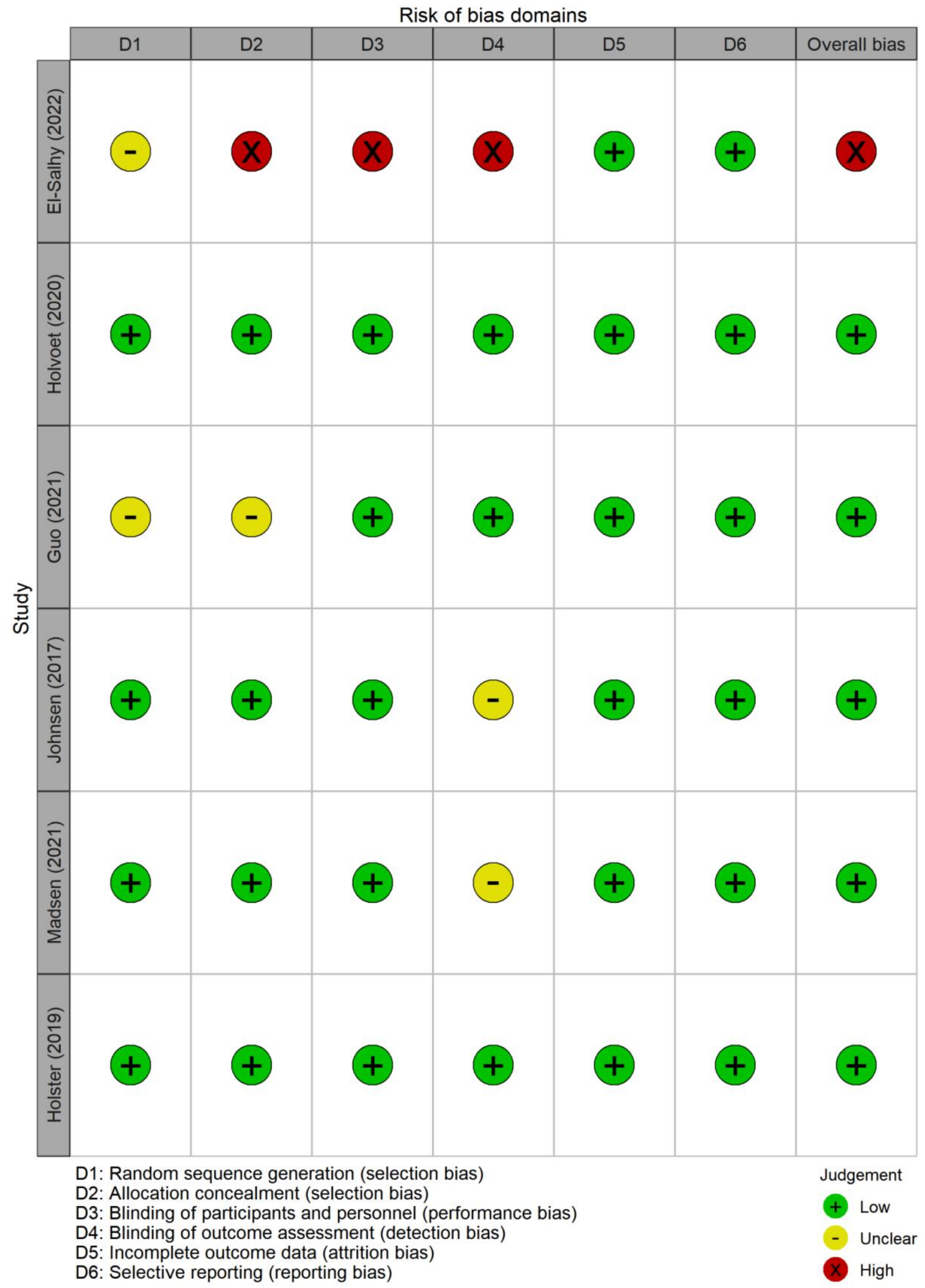

Supplementary Figure S9 Traffic light plot of study-by-study bias assessment for 6 included RCTs of FMT versus placebo in irritable bowel syndrome: effect on global symptom change from baseline using the Cochrane Collaboration's risk of bias tool.
